# A Bulk Segregant Gene Expression Analysis of a Peach Population Reveals Components of the Underlying Mechanism of the Fruit Cold Response

**DOI:** 10.1371/journal.pone.0090706

**Published:** 2014-03-05

**Authors:** Clara Pons, Cristina Martí, Javier Forment, Carlos H. Crisosto, Abhaya M. Dandekar, Antonio Granell

**Affiliations:** 1 Plant Genomics and Biotechnology lab, Instituto de Biología Molecular y Celular de Plantas, Consejo Superior de Investigaciones Científicas, Universidad Politécnica de Valencia, Valencia, Spain; 2 Department of Plant Sciences, University of California Davis, Davis, California, United States of America; Institute of Genetics and Developmental Biology, Chinese Academy of Sciences, China

## Abstract

Peach fruits subjected for long periods of cold storage are primed to develop chilling injury once fruits are shelf ripened at room temperature. Very little is known about the molecular changes occurring in fruits during cold exposure. To get some insight into this process a transcript profiling analyses was performed on fruits from a PopDG population segregating for chilling injury CI responses. A bulked segregant gene expression analysis based on groups of fruits showing extreme CI responses indicated that the transcriptome of peach fruits was modified already during cold storage consistently with eventual CI development. Most peach cold-responsive genes have orthologs in *Arabidopsis* that participate in cold acclimation and other stresses responses, while some of them showed expression patterns that differs in fruits according to their susceptibility to develop mealiness. Members of ICE1, CBF1/3 and HOS9 regulons seem to have a prominent role in differential cold responses between low and high sensitive fruits. In high sensitive fruits, an alternative cold response program is detected. This program is probably associated with dehydration/osmotic stress and regulated by ABA, auxins and ethylene. In addition, the observation that tolerant siblings showed a series of genes encoding for stress protective activities with higher expression both at harvest and during cold treatment, suggests that preprogrammed mechanisms could shape fruit ability to tolerate postharvest cold-induced stress. A number of genes differentially expressed were validated and extended to individual genotypes by medium-throughput RT-qPCR. Analyses presented here provide a global view of the responses of peach fruits to cold storage and highlights new peach genes that probably play important roles in the tolerance/sensitivity to cold storage. Our results provide a roadmap for further experiments and would help to develop new postharvest protocols and gene directed breeding strategies to better cope with chilling injury.

## Introduction

Most of what we currently know about how plants cope with low temperatures stems from the work carried out in the temperate model plant *Arabidopsis*, where it has been studied in vegetative tissues in relation to cold acclimation [1], a process integrated with developmental programs that results in extensive transcriptome and metabolome reorganization which appears to act, at least in part, to protect membranes and proteins against the severe dehydration stress that occurs during freezing [2]. By using mostly seedlings, the regulatory factors influencing the expression of cold regulated (COR) genes have been identified [3]. Three cold-induced transcriptional regulatory factors known as C-repeat binding factor (CBFs) [4] control the expression of a major regulon of COR genes to confer plant freezing tolerance [5], and may play a role in chronic low temperature adaptation [6]. Upstream of the CBF regulatory hub, two cold-sensing pathways have been described. One involves ICE1(inducer of CBF expression 1) [7]. The other involves calcium and the calmodulin binding transcription activators CAMTA3 and CAMTA1 [8]. In addition, some important components mediating cold and freezing tolerance through CBF-independent pathways has been described [9], [10], [11]. Besides transcriptional regulation, there are evidences which indicate that cold acclimation is also regulated at the chromatin [9], post-transcriptional [12], [13], translational [14]and posttranslational levels [15], [16]. Further, ABA-independent and -dependent pathways regulate cold-responsive genes, and ABA acts synergistically with the cold signal [17]. Although much attention has been paid to ABA in relation to the cold response [18], there is growing evidence that other hormones such as auxins, brassinosteroids, ethylene, jasmonic acid and salicylic acid are involved in cold acclimation [19], [20], [21], [22], [23].

In general, basic cold responses can be shared among different plant species [24] and organs [25], although, some structural and regulatory differences have been observed between tolerant and sensitive plant species [26], [27]. In fruits, however, cold might have an impact on a subset of specific characteristics and eventually affect ripening [28]. Apple and some pear cultivars require cold acclimation to set up ripening [28], [29]. In apple, a CBF like gene promotes softening in absence of ethylene and, probably, cold and ethylene act independently and synergistically with each other to induce fruit softening [28].

Little is known about low temperature responses in summer fruits such as peach because the chilling period occurs naturally as winter cold comes, when plants have not yet fruits. The horticultural industry uses similar temperatures to those triggering cold acclimation to preserve fruit quality after harvest. Despite widespread use, this technology has its restrictions, as many fruits and vegetables are sensitive to low temperatures and develop a syndrome named chilling injury (CI) [30]. Peach fruits subjected to long cold storage periods can develop a form of CI called mealiness/woolliness, a flesh textural disorder characterized by a lack of juiciness [31], which appear only after fruits have shelf-ripened at room temperature [32]. Peach exhibits a high degree of genetic variability for chilling tolerance, with the most sensitive cultivars being damaged after 1 week of cold storage and the most tolerant remaining undamaged for at least 5 weeks [32]. Genetic analysis indicates that chilling injury in peach is a quantitative trait, and a number of QTLs for chilling injury have been mapped to the peach genome [33], [34], [35], [36]. Most of the reports on mealiness emphasize the changes in the cell wall during shelf life ripening after cold storage [37], [38], [39]. Recently, large-scale approaches have identified new peach genes associated to mealiness during shelf life [40], [41], [42], [43]. Nevertheless, the information about what happens during cold storage is scant. During cold storage physiological alterations has been described. Firmness and ethylene production were reduced [41], [44]. Alterations in cell wall transcriptome, enzyme activity and in cell wall polymers metabolism still occur during cold storage, which affect in the manner fruits ripened during subsequent shelf life [39], [41]. Further, stress responsive genes increase during cold storage [41], [42] while genes related to energy metabolism decrease [42]. Unfortunately, these reports did not go deep in the analysis of the genes and their functions and, failed to associate gene expression to chilling sensitivity as they were based in the response of a single genotype subjected or no cold. Contrasting genotypes can serve as a powerful tool for understanding the physiological and molecular mechanisms of chilling tolerance in peach. In a preliminary study the expression of ten cold induced genes was associated to the tolerance to chilling injury [45]. More recenty, Dagar et al. [46]] identified a group of differentially expressed genes between two varieties at harvest, which are probably related to their tolerance or susceptibility to develop CI.

In this study, we have used an adaptation for the gene expression data of the Bulked Segregation Strategy [47], [48]; dubbed herein as the Bulked Segregant Gene Expression Analysis (BSGA). We used the custom cDNA Chillpeach microarray [45] as expression profiling platform on RNA from pools of fruits from siblings of the Pop-DG population [49] exhibiting extreme cold responses. Our approach was validated and extended to a number of individual members of the population with different degrees of cold susceptibility by using medium throughput Fludigm RT PCR.

## Methods

### Plant Material and CI/MI Measurements

Siblings from Pop-DG mapping population [49], segregating for chilling injury, were used in this study. Mesocarp samples from fruits of the following Pop-DG siblings were used: 49/59, 84/85, 86/87 and 132/133 with high sensitivity to mealiness (S) and 71/72, 88/89, 134/135, 142/143 with low sensitivity (LS). These Pop-DG siblings with similar horticultural characteristics but with extreme differences on mealiness development were selected because their sensitivity phenotype was consistent for 3 years prior this study (Fig. S1 in File S1). In all cases, fruits were harvested at the mature commercial stage (M) according to Kader & Mitchel [50] with flesh firmness of 12–14 lb, soluble solid content (SSC) of 11–14% and tritrable acidity (TA) of 0.5–0.7% (Table S1). A group of 12 fruits M were directly allowed to ripen at 20°C to the edible firmness of 2–3 lb (R samples) as controls. For cold treatments, M fruits were forced-air cooled at 0–2°C within 6 h of harvest and were then stored at 5°C with 90% relative humidity for 1, 2 and 3 weeks (CS samples). Chilling injury of each sibling after the cold storage period was expressed as Mealiness index (MI), i.e the proportion of measured fruits with mealiness when ripened for 2–3 days at 20°C. Mealiness was assessed as the percentage of free juice content accordingly to Campos-Vargas et al. [44] using the quantitative method described by Crisosto et al. [51]. Fruits shelf ripened after one week of cold storage were checked for other chilling disorders (flesh bleeding and flesh browning) as in Martinez-Garcia et al. [52]. The samples representing at least 6 fruits from each genotype and treatment were bulked and immediately frozen in liquid nitrogen before storing at −80°C until they were used for RNA isolation.

### Microarray Hybridization, Scanning and Data Pretreatment

For the microarray experiments, equal amounts of RNA from each genotype in a given control or treatment group were mixed in the corresponding S and LS pools. The RNA pools were all hybridized using the ChillPeach microarray [45]. All samples were compared using a dye-swap design against a common superpool reference, composed of equal amounts of RNA obtained from all the mesocarp samples. Three replicates from each sample pool were hybridized in each case, one of them dye-swapped.

RNA purification, sample preparation and hybridization to Chillpeah microarray were performed as described in Ogundiwin et al. [45]. Intensity values were obtained as the median of ratios using GenePix 4000B scanner (Axon Instruments). Data files were imported into Acuity 4.0 (Axon Instruments) for normalization and analysis. Only spots with intensity values higher than the background plus two standard deviations of the background median, in at least one channel, were used for analyses. Before normalization, the median local feature background was subtracted. Data were normalized by Lowess (locally weighted scatter plot smoothing) with a centered print-pin tip using the Acuity default values. To generate the raw data to be used for the expression analysis, a Lowess M Log Ratio was used as the expression value, and patterns with more than 80% of non missing values were selected. In all, 3350 probes (78.62∼% of the ChillPeach probes) met the threshold for hybridization quality (Table S2). The data sets supporting the results of this article are available in the Array express repository, [E-MEXP-3902].

### Expression Analysis

Differentially expressed genes were identified from the raw dataset using the Significance Analysis of Microarray software [53]. Missing values were imputed by 10-Nearest Neighbors Imputer algorithm, with 100 blocked permutations and a random seed value set by default in the program. PCA and 2D-hierachical cluster analyses were performed on the significant data using Acuity (Axon instruments). A principal component analysis (PCA) was calculated for those factors explaining 100% of variance. For calculations spots with missing values were replaced with the average values across the arrays. Profiles with the same shape pattern were centered around the mean value across arrays, to avoid the effect that the magnitude of response might have on the average profile. For the hierarchical cluster, a Pearson correlation centered on 0 was used as a similarity metrics. A complete linkage was used to link clusters together to produce the tree. Transcripts and/or samples were ordered in the clusters according to their contribution to principal component 1 of the PCA performed with the same dataset. The ChillPeach genes were classified into 34 distinct functional categories and 702 specific processes (Table S3) by extensively reviewing the literature and by searching in reference databases (Methods S1). Functional enrichment on a ranked list of genes was performed with, a local, customized version of ‘catscore.pl’ Perl script described in Cheung et al. [54], using a two-tailed Fisher exact t-test with adjusted *p*-value cut-off of 0.05.

### Correlation Analysis between Transcript Levels and Degree of Mealiness

Correlations were calculated by the Pearson product moment correlation method using Matlab 2007 (The MathWorks, Inc.). *P* values below 0.01 were selected for statistical significance. A statistical significance level of 1% was assessed with the correlation coefficients over 0.8. Those genes whose expression profiles contained 100% of data points in the samples analyzed were used to calculate correlations. The complete list of the microarray-wide gene expression correlations with the Mealiness Index (MI) are listed in Table S3. Functional enrichment is performed as indicated above.

### A Medium-throughput Quantitative RT-PCR Analysis Using a Dynamic Array by Fluidigm

The 96.96 dynamic arrays were obtained from the Fluidigm Corporation and were used to set up four sets of qRT-PCR reactions of 64 cDNA preparations corresponding to 32 samples: 15 genotypes in the M stage and/or CS1 samples and 5 pools (M-S, M-LS, CS1-S, CS1-LS and the reference superpool used for the microarray analyses). Two biological replicates were included in each array for all the 15 genotypes and pools, each one representing at least three different fruits. Two replicated 96.96 Fluidigm dynamic arrays were used.

For the Fluidigm analysis, 72 genes were selected from our microarray results (Table S7). Oligo pairs for selected genes were obtained using the Primer Express version 2.0 software (Applied Biosystems). To design primers, the following conditions were used: Tm 58–60°C, GC content 20–80%, primer length 20–22 base pairs and an amplicon size of 140–150 bp. A virtual PCR was carried out for each oligo pair obtained with the ‘primersearch’ program from the EMBOSS open software suite [55], using the full set of known peach sequences as potential template sequences. The interrogated peach sequence databases included the ChillPeachDB [45], ESTreeDB [56] and GDR_Prunus [57] sequences. Only the oligo pairs yielding a single PCR product from each unique gene, based on the sequence assembly of all the known *Prunus* sequences, were considered. When more than one specific oligo was obtained for a gene, the oligo pair with the lowest penalty value (as provided by the Primer Express version 2.0 software for oligo identification), and which mapped most of the 3′ end of the gene, was selected using custom Perl scripts.

Three genes were selected to normalize qRT-PCR results on the basis of low variability in the chillpeach microarray under all conditions analyzed in this paper: a gene with unknown function (PPN036E09), an ABC1 family protein(PPN076G09) and, an esterase/lipase/thioesterase gene (PPN078E12) They were validated by qRT-PCR as described in [45]). The comparative ΔΔCt method, as described by in Livak and Schmittgen [58], was used to confirm a flat pattern throughout the samples.

For the Fluidigm analysis, the cDNA synthesized from total RNA following standard methods was diluted to 1∶10 using the DA Assay Loading Buffer (Fluidigm). The Nanoflex 4-IFC Controller and the BioMark Real Time PCR system by the Fluidigm Corporation were used to run the dynamic arrays under the standard conditions employed at the General Hospital lab, Valencia, Spain. The cycling program consisted of 10 min at 95°C followed by 40 cycles of 95°C for 5 sec and 1 min at 60°C.

The relative gene expression values were determined using PerlqXpress (manuscript in preparation). PerlqXpress was used to calculate “fold expression values” (FC) from the Ct values obtained directly from the BioMark Real-Time PCR Analysis Software (Fluidigm). Briefly, PerlqXpress filter outliers within a sample, corrected differences in background control levels, centers and scales data. The mean centered and scaled Ct values were transformed into relative quantities (RQ) using the exponential function with the efficiency of PCR reaction as its base. For each gene the RQ was corrected using a normalization factor. FC is calculated by dividing normalized RQ to reference sample in each biological replicate (in this case reference pool used in the microarrays). Mean, standard deviation, and coefficient variation were calculated for each replicate. Replicates were filtered by the coefficient variations. At least 4 good replicates were used to calculate “fold expression change” values.

To extend the validity of the results obtained in the pools to individual lines, for which we had individual MI index values, qRT-PCRs were performed on 15 individual peach genotypes from the popDG progeny (Table S8). For each gene pair in a predefined expression set, the Pearson correlation coefficients between their expression profiles in the individual Pop-DG siblings were obtained by Gitools 1.8.2 [59]. A gene was selected as consistent and was confirmed over the individual lines when it correlated with a predefined expression pattern.

## Results

### Differential Cold Response to Chilling Temperature in the Fruits of the Pop-DG Peach Population

**Figure 1:**
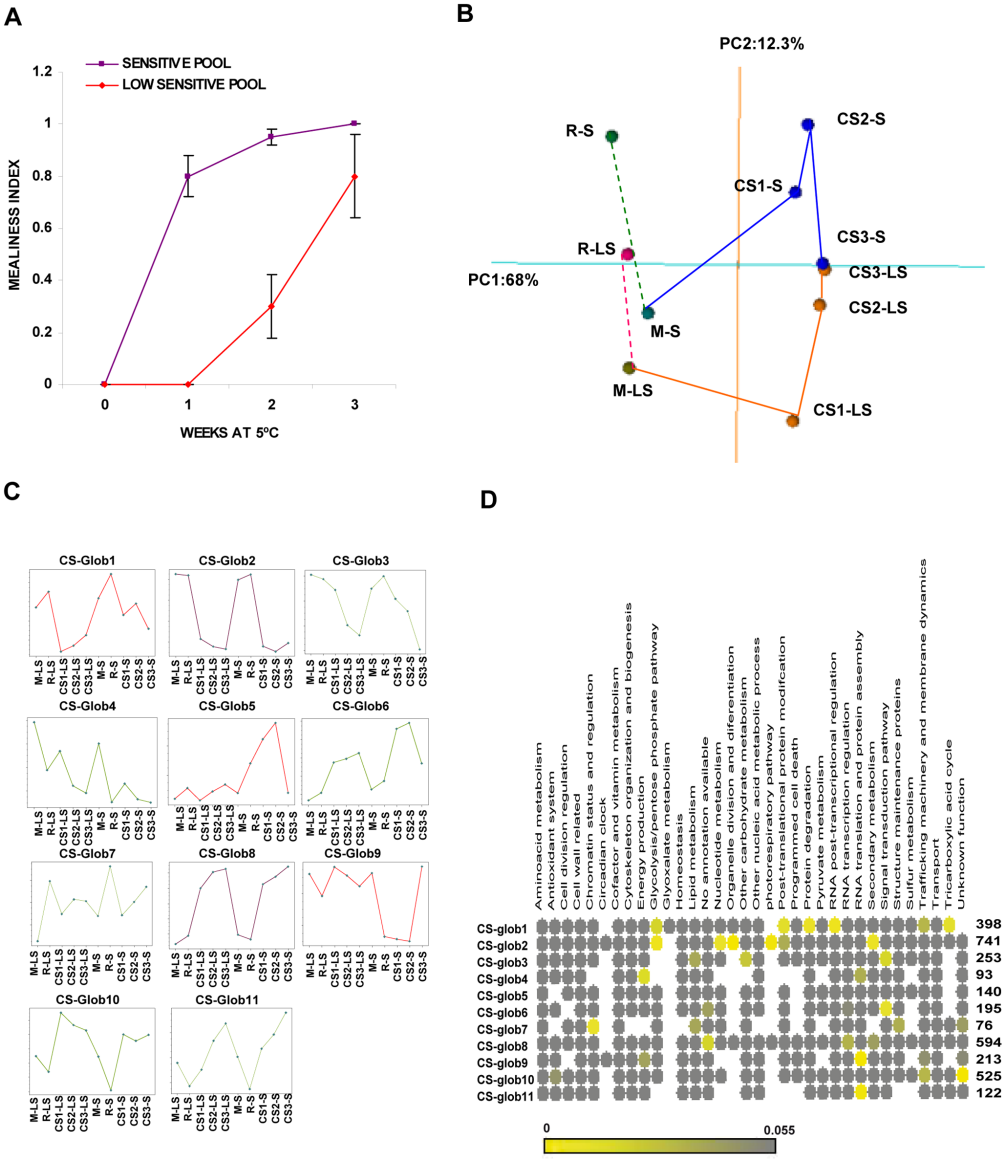
Mealiness index of pools of peach Pop-DG siblings and global gene expression analysis of Chillpeach transcripts in response to cold storage. A) Average mealiness index (MI) of pools S and LS from fruits shelf life ripened for 2–3 days at 20°C after being stored for up to 3 weeks at 5°C; B) Principal Component Analysis (PCA) of the global expression profile showing the most variation of each treatment condition (averaged from three replicates). First principal component (PC1) is shown on x-axis while the second principal component (PC2) is shown on y-axis. C) Clusters resulting from the unsupervised two-dimensional hierarchical clustering (Fig. S2). Y-axes represent the normalized expression ratio (Log2 M) of three biological replicates in relation to a reference pool. Red represents qualitative differences, purple depicts the genes regulated in a similar manner and green refers to the genes showing quantitative differences between the LS and S pools. D) The functional categories overrepresented in each cluster (Fig. 1C) are shown as a heatmap obtained with matrix2png. Enriched functional categories with Fisher test p-values <0.05 are colored in grades of yellow. The number of genes in each cluster is indicated to the right of the heatmap. M = mature fruits, R = mature with 2–4 days ripening at 20°C, CS1 = M +1 week cold storage at 5°C, CS2 = M +2 weeks cold storage at 5°C, CS3 = M +3 weeks cold storage at 5°C.

Harvest maturity, a factor known to influence mealiness [60], was tested before cold treatments to ensure all fruits were in the same maturity stage. Table S1 shows there were no significant differences in firmness, SSC and TA between genotypes. This indicates that at harvest, both populations were at the same physiological stage and differences in the subsequent cold response can be mainly attributed to the cold sensibility without significant distortions owing to lack of adequate maturity stage. To asses the effect of the cold stress on peach fruits from siblings of the Pop-DG population, a subset of the cold stored fruits were ripened for 2–3 days at 20°C and mealiness was evaluated as the proportion of measured fruits with mealiness or Mealiness index (MI). Figure 1A shows the average MI of pools of fruits grouped according to their sensitivity to develop mealiness. The pool S had higher MI as compared with pool LS after the same cold storage times (Fig. 1A), although tend to converge after increasing cold storage periods, indicating a non complete (but clear with huge market importance) tolerance of fruits LS. The difference was more pronounced after one week of cold storage at 5°C, where the mealiness symptoms were already visible in the pool S but not the pool LS (Fig. 1A). No significant differences in the frequency of other CI symptoms were observed between pools S and LS (Table S1). Thus the characteristic feature, differentiating the cold response of the pools, was their sensitivity to develop mealiness. Given that the proportion of mealy fruits increased with the time of cold storage, our hypothesis is that despite mealiness was not showing until fruit was allowed to ripe [32], relevant molecular changes may had already started to occur during cold storage.

### A Global Non Target Analysis of the Transcript Profiles in the Pop-DG in Response to Cold

Bulked segregant analysis [47], [48] in combination with the Chillpeach expression microarray [45] was used to compare the transcriptomes of peach fruits from the S and LS Pop-DG siblings. In all, 3350 transcripts (Table S2) showed a significant difference in expression levels at least for one condition (samples M, R and CS for pools S and LS) using two criteria: a false discovery rate (FDR) <5% and a p-value <0.05. The principal component analysis (PCA) of the complete dataset variance is seen in Figure 1B. PC1 (68% variance) clearly separated fruit samples which came directly from cold storage (CS), from fruits M and R (Fig. 1B). The proximity between fruits M and R, if compared to CS, indicated that the effect of cold storage on the peach transcriptome was much greater than that of ripening. PC2 (12% of variance) separated fruits M from R. Both, fruits S and LS seemed to follow parallel ripening programs, but fruits LS showed delayed or less intense ripening transcriptomic changes than fruits S. It should be noted that Pop-DG siblings in each pool were selected on the basis of their cold response, as revealed by the MI after shelf life ripening, so it is not surprising that some differences in the ripening programs may exist. In addition, PC2 roughly separated cold stored samples according to the eventual increase in the MI of the fruit should they be submitted to shelf life after cold storage (Fig. 1A and B). According to this component, fruits from the pool S stored for 1 or two weeks have achieved a pattern of ripening similar to fruits R (as they had similar values in PC2). This may indicate that during cold storage at 5°C, some internal ripening may result in chilling sensitivity and in a shortened shelf life. The loading plots for PC2 (i.e., the contribution of each gene to the separation by a given principal component) revealed 37 genes among with were genes previously reported in the regulation of temperature responses, including the transcriptional factor CBF [4], GASA5 [61] and SCR2 [20] (see Table S3). Thus the transcript levels contributing to component PC2 may be relevant for the development of a tolerance mechanism in cold, which could affect the way cold storage interrupted or slowed down the ripening program and eventually how fruits ripen afterward.

The bidimensional hierarchical cluster (2D-HCA) analyses revealed a similar sample separation to that obtained with PCA (Fig. S2 in File S1). Furthermore, 2D_HCA segregated CS1-LS from the rest of the cold-stored fruits (Fig. S2 in File S1), according to the fact, that if fruits CS1-LS ripen, they do not develop mealiness. These results indicate that from the molecular point of view one week of cold storage is a critical time with maximum differences expected to be found at this stage between fruits S and LS, including any CI associated trait. This global analysis also revealed that, although the expression profiles were generally similar between the S and LS pools of fruits, there were qualitative and quantitative differences (i.e., the kinetics or levels reached, or both). To further describe the cold response mechanism from a global point of view and its possible relation to eventual CI, we conducted a functional enrichment analysis (Fig. 1D and Results S1) of the 11 resulting clusters from 2D-HCA (Fig. 1C). The most overrepresented functional category in cluster CS-glob8, containing genes up-regulated during cold storage in both fruit pools, was *RNA transcription regulation*, which comprised 47 genes (Fig. 1D and Results S1). In this category, we found several transcription factors whose orthologs were up-regulated during cold acclimation in *Arabidopsis* and some were assigned to specific cold acclimation regulons (Table 1; for references see Table S4). The other functional category enriched in CS-glob8 was with 37 genes, *secondary metabolism,* a functional category previously associated with cold tolerance [62], [63]. In addition, and in agreement to the higher tolerance of fruits LS, *structure maintenance proteins* and *an antioxidant system* were among the functional categories overrepresented in differential clusters CS-glob7 and CS-glob 10 (Fig. 1D), both highly induced in the pools of fruits LS as compared to fruits S (Fig. 1C). Moreover, cluster CS-glob 9 was enriched in *RNA translation and protein assembly, energy production,* and *trafficking machinery and membrane dynamics* (Fig. 1C and 1D), indicating that these activities can be enhanced in fruits LS. This suggests that some kind of cold adaptation was activated in both S and LS peach fruits during cold storage.

**Table 1:** Transcription Factors genes common Cold-Upregulated in Peach with Stress and Hormone Related Roles.

Transcriptionfactorfamily	ChillpeachID	Gene description	Arabidopsis GeneSymbol	stress/hormone	Coldregulon
**AP2/EREBP**	PPN039F03	Putative dehydration-responsive elementbinding protein	RAP2.4	CA-DR, drought, light, ethylene	
	PPN078E06^a^	EREBP-4 like protein		CA-DR	
**AUX/IAA**	PPN046H05	Auxin-responsive protein IAA13	IAA13	AUX negative regulation	
**C2C2-CO-like**	PPN075B03	zinc finger (B-box type) family protein	STH2	CA-DR, light	
**C2H2**	PPN046D02	Zinc finger protein 4	ZFP4	CA-DR	
	PPN053C05	Zinc-finger protein 1	AZF2	CA-UR	
**GRF**	PPN044H02	14-3-3 protein 3	GRF2	CA-DR	
**HD-ZIP**	PPN047H02	Homeobox-leucine zipper protein HAT22	HAT22	drought, light, carbon sensing	
**HSF**	PP1002D06	Heat shock factor	HSFB1	high up-regulated in Arabidopsis*chs* mutants	
	PPN001A09	Heat shock factor	HSFB1	high up-regulated in Arabidopsis*chs* mutants	
	PPN054G07^a^	Heat shock factor	HSFB1	high up-regulated in Arabidopsis*chs* mutants	
	PPN055B05	Similarity to heat shock transcription factor	HSFC1	CA-UR	ICEI
	PPN077H06	Heat shock transcription factor	AT-HSFA4A	CA-UR, high up-regulated in *hos15*mutants	HOS15
**MADS-box**	PPN004D05	MADS box transcription factor	SVP/AGL22	CA-UR	
	PPN058B02	MADS box transcription factor	AGL24	cold up-regulated (vernalization)	
**MYB**	PP1006F11	MYB1	ATMYB6	CA-DR	
**NAC**	PP1001F06	NAM-like protein	ATNAC2/anac056	CA-DR	
	PPN054B06	No apical meristem protein-like	anac073/SND2	CA-DR	
	PPN073C10	NAM-like protein	anac083/VNI2	CA-DR, ABA-mediated abiotic stress	
**PHD**	PPN035F03	hydroxyproline-rich glycoproteinfamily protein	EDM2	defense to pathogens	
	PPN051C10	ABI3-interacting protein 2	AIP2	CA-UR	ICE1
**TUB**	PPN066C05	Tub family, putative	AtTLP1	CA-UR	
**WRKY**	PPN001D05	DNA binding protein WRKY2	WRKY3	CA-DR	

a positive correlation with projected MI.

Arabidopsis response during cold acclimation:. CA-UR cold acclimation up-regulated, CA-DR, cold acclimation down-regulated.

To see references supporting the involvement of these genes in stress and/or hormones see Table S3.

The genes in cluster CS-glob 2 were down-regulated in both S and LS fruits (Fig. 1C), and were enriched in *glycolysis/pentose phosphate pathway, the photorespiratory pathway* and *organelle division* (Fig. 1D). Lowered expression levels of carbohydrate metabolism and glycolytic genes correlated to cold sensitivity in Arabidopsis [62]. However, the extensive down-regulation of primary metabolism, together with the down-regulation of *posttranscriptional, posttranslational and protein degradation* (see cluster CS-glob 1 in Fig. 1C and 1D), was probably associated with the relative higher cold tolerance of fruits LS.

### Stage-specific Changes in the Transcript Program Associated with the Differential Cold Response

**Figure 2:**
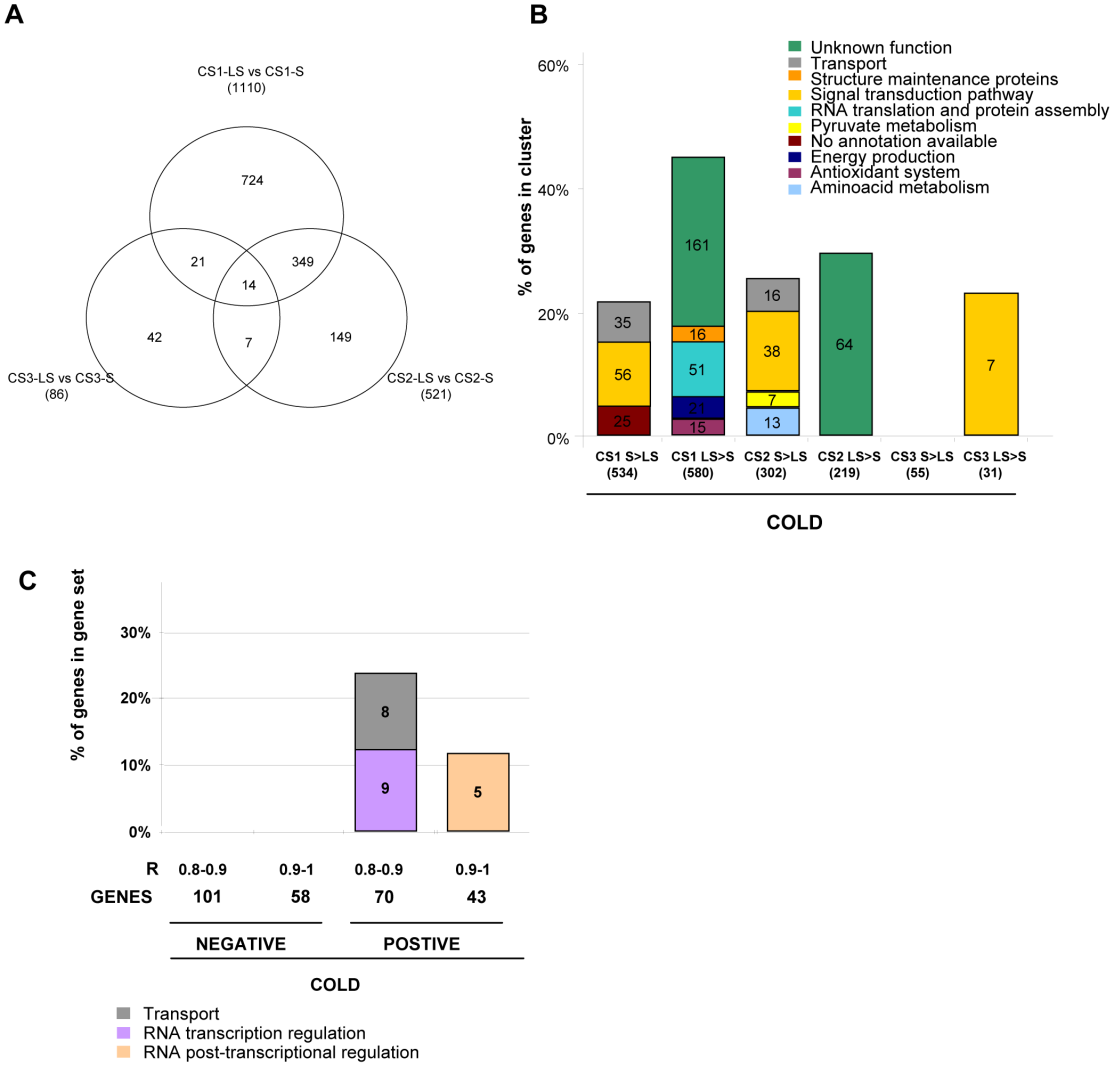
Differential gene expression between the S and LS fruit across the cold storage CS series. A) A Venn diagram depicting the differentially expressed genes (FDR<0.05 and q-value<0.05) between tolerant and sensitive fruit at each time of cold storage. B) The over-represented functional categories (p-value 0.05) corresponding to the differentially expressed genes between the LS and S pools at each time of cold storage. C) The functional categories enriched in the genes whose expression profiles correlated with the projected MI fruits should have when shelf life ripened. Pearson: 1<r <0.9 and 0.9< r 0.8. M = mature fruits, R = mature with 2–4 days ripening at 20°C, CS1 = M +1 week cold storage at 5°C, CS2 = M +2 weeks cold storage at 5°C, CS3 = M +3 weeks cold storage at 5°C.

A direct one-to-one comparison was made between the transcriptomes of the samples S and LS at the same time of cold storage, given the notion that this analysis would outperform the general profile comparison to identify the candidates to be involved in tolerance/susceptibility to cold (before obvious injury symptoms appear). Figure 2A shows how the number of differentially expressed genes at each time decreased with storage time (Fig. 2A), thus confirming PCA results (Fig. 1B). Functional enrichment analysis (Fig. 2B, Results S2) showed that by 1 week of cold storage, the transcripts with higher levels of expression in fruits CS1-LS were preferentially related to *energy production, RNA translation and protein assembly, the antioxidant system, structure maintenance, and genes with unknown functions* (for more details, see Table 2 and Table S3). Those transcripts with lower levels in LS fruits (and therefore higher levels in S fruits) were enriched in *signal transduction elements and transport* (Fig. 2B and Table 3). As 1 week cold storage is critical timing i.e. when maximum differences were shown when later transferring fruits to shelf life ripening (Fig. 1A–B), these functions may play a prominent role in the tolerant/sensitive character of fruits (for more details of these genes, see Table 3 and Table S3). By 2 weeks of cold exposure, only the genes with unknown functions were overrepresented in the tolerant pool (Fig. 2B and Results S2), whereas a significant enrichment was noted for the genes linked to *amino acid metabolism, pyruvate, signal transduction and transport* in the genes at higher levels in CS2S. Interestingly, most of the genes expressed at higher levels in S fruits by 2 weeks had already reached this state by 1-week of cold storage (Table S3). As two weeks of cold exposure results in mealiness upon shelf life in both S and LS fruits (Fig. 1A), but with large differences in MI severity, high levels of these genes may correlate negatively with the tolerant character of fruits. After 3 weeks in the cold, only the highly expressed genes in tolerant fruits showed *signal transduction* as an overrepresented class (Fig. 2B and Results 2). In this case, the genes differed from those identified as being overrepresented at 1 and 2 weeks (Table S3). At this time, both S and LS developed mealy fruits with MI 1.0 and MI 0.8, respectively (Fig. 1A), but S was probably much more severely affected or underwent other downstream processes.

**Table 2:** Expression Regulators and Signaling Elements with High Expression in Low Sensitive Fruits at One Week of Cold Storage with stress and hormone related roles.

Functional category	ChillpeachID	Gene description	Arabidopsis GeneSymbol	HCApattern	stress/hormone	Coldregulon
***RNA post-transcriptional regulation***						
**RNA biogenesis and** **processing**	PPN035E09	Dehydration-induced proteinERD15	ERD15	CS-glob9	negative regulator ABA	
	PPN048C02	Sm-like protein	SAD1	CS-glob10	negative regulator ABA	
***RNA transcription regulation***						
**AP2/EREBP family**	PPN049D05	similar to DREB3		CS-glob8	cold, drought, salinity	
	PPN054B03	CBF1	DREB1A/CBF3	CS-glob9^a^	CA-UR, AUX down-regulated	ICE1/CBF
**AUX/IAA family**	PP1009D02	IAA16 protein	AXR3/IAA17	CS-glob9	negative regulator in AUXand ABA signaling	
	PPN057F01	AUX/IAA protein	PAP2/IAA27	CS-glob4	light	
**b-ZIP family**	PPN049B04	BZIP transcription factorbZIP68		CS-glob10	light, cold	
**C2C2-CO-like Family**	PPN050G11	zinc finger (B-box type) familyprotein	AT4G27310	CS-glob10	cold	AREB/ABF
**CAMTA family**	PPN075B05	Anther ethylene-up-regulatedprotein ER1	SR1	CS-glob2^b^	cold up, salinity, defenseand ET	
**CCAAT Family**	PPN006E07	Repressor protein	NF-YB13	CS-glob10	darkness	
**HMG-family**	PPN042B12	HMG-protein	HMGB1	CS-glob2^b^	stress	
**MYB-family**	PPN041A07	myb family transcriptionfactor	CDC5	CS-glob9	defense responses, light,cold	
	PPN055C11	Sucrose responsive elementbinding protein	ATMYBR1/ATMYB44	CS-glob10	cold	ICE1
**PHD-family**	PPN051C09	PHD finger protein At5g26210	AL4	CS-glob3	cold, salinity and ABA	
	PPN068F05	PHD finger protein At5g26210	AL4	CS-glob10	cold, salinity and ABA	
**RNA transcription** **machinery**	PPN027A09	Sigma-like factor precursor	ATSIG5	CS-glob2	light	
***Protein degradation***						
**Proteolysis control-Signalosome**	PPN042D08	COP9 signalosome complexsubunit 8	COP9	CS-glob8	light	
***Signal transduction pathway***						
**ABA signaling/reversible protein** **dephosphorylation**	PP1009B12	Protein phosphatase 2C	ATPP2CA/AHG3	CS-glob10	negative regulator ABA	
	PPN029F02	Protein phosphatase 2C(AtP2C-HA)	HAB1	CS-glob3	negative regulator ABA	
**Aux signaling/Unknown** **SAUR protein**	PP1001B04	expressed protein (DUF298)	AAR3	CS-glob4^b^	AUX response regulation	
	PPN015D06	auxin-responsive familyprotein,(SAUR)		CS-glob4	AUX	
	PPN051E05	auxin-responsive familyprotein,(SAUR)		CS-glob2	AUX	

a contribution to PC2 (Fig. 1A) negative;

b negative correlation with projected MI.

Arabidopsis response during cold acclimation: CA-UR cold acclimation up-regulated.

To see references supporting the involvement of these genes in stress and/or hormones see Table S3.

**Table 3:** Expression Regulators and Signaling Elements with High Expression in High Sensitive Fruits at One Week of Cold Storage with stress and hormone related roles.

Functional description	ChillpeachID	Gene description	ArabidopsisGene Symbol	HCApattern	CS1 SvsTpattern	stress/hormone
***Energy production***						
**vacuolar ATP production and** **cytoplasmic pH** **regulation**	PPN014F01	Vacuolar H+-ATPase subunit C	DET3	CS-glob5	S>LS	Light, AUX, ABA
***Protein degradation***						
**chloroplast protease**	PPN022B02	ERD1 protein, chloroplastprecursor	ERD1	CS-glob1	S>LS	ABA, drought, salinity,dark induced senescence
**peptidase**	PPN007E05	aminopeptidase M, similar	APM1	CS-glob1	S>LS	AUX transport regulation
**Proteolysis control-Signalosome**	PPN008B05	COP9 signalosome complexsubunit 2	FUS12/ATCSN2	CS-glob1	S>LS	light
**SCF complex assembly and** **disassembly**	PPN068H05	Putative TIP120 protein	CAND1	CS-glob1	N/A	AUX signaling
**Ubiquitin ligase E3 complex/** **SFC-culin**	PPN030D09	Cullin	AXR6/CUL1	CS-glob1	S>LS	AUX signaling regulation,light
	PPN032E01	Cullin family	CUL3	CS-glob1	N/A	ET production, light
***RNA transcription regulation***						
**ARF-family**	PPN051B02	Auxin response factor 2	NPH4/ARF7	CS-glob5	N/A	AUX response regulator,cold
	PPN072B07	Auxin response factor 5	MP/ARF5	CS-glob7	S>LS	AUX signaling andtransport regulator
**b-HLH family**	PPN080F10	Prf interactor 30137	LHW	CS-glob6	S>LS	AUX signaling
**GRAS-family**	PPN078C08	GRAS1	SCL14/GAI	CS-glob8	S>LS	CA-UR
**GroTLE transcription corepressor** **family**	PPN076D05	Transcriptional corepressorLEUNIG	LUG	CS-glob1	S>LS	AUX signaling regulator
**HB-family**	PPN069A12	BEL1-like homeodomaintranscription factor	BLH1	CS-glob5	S>LS	drought, salinity
**MADS-box family**	PP1009H08	MADS box transcription factor	AGL24	CS-glob8	S>LS	cold up-regulated(vernalization)
**MYB-family**	PPN058F01	GAMYB-binding protein	SKIP1	CS-glob1	S>LS	ABA, drought, salinity
**NAC-family**	PPN023B05	NAC domain-containing protein78	NAC2/anac078	CS-glob1	S>LS	AUX, ET, salinity
	PPN062G07	NAC family protein	ATAF1	CS-glob2	S>LS	ABA, drought, salinity,pathogen
**RNA transcription machinery**	PPN067A07	Elongator component	ELO1	CS-glob1	N/A	ABA, AUX
	PPN070H08	C-terminal domain phosphatase-like 2	CPL2	CS-glob6	S>LS^a^ **^,b^**	osmotic (salinity) stressand AUX responses
**Unknown transcription coactivator**	PPN063D04	COP1-Interacting Protein 7	CIP7	CS-glob2	S>LS	light
***Secondary metabolism***						
**Aux metabolism/Aux** **biosynthesis**	PPN034D04	Flavin-containing monooxygenase,putative	YUC10	CS-glob8	S>LS	AUX biosynthesis
**Aux metabolism/Aux** **conjugation**	PPN030D12	similar to Putative auxin-amidohydrolase precursor		CS-glob5	S>LS	AUX metabolism
**Aux metabolism/Aux** **deconjugation**	PPN017F04	Auxin and ethylene responsiveGH3-like protein	GH3.1	CS-glob1	S>LS	stress, AUX metabolism
**Carotenoid metabolism**	PP1005H08	Zeaxanthin epoxidase, chloroplastprecursor	ABA1	CS-glob8	S>LS	ABA biosynthesis
**Ethylene biosynthesis**	PP1009G10	1-aminocyclopropane-1-carboxylateoxidase	EFE/ACO4	CS-glob2	S>LS	ET biosynthesis
***Signal transduction pathway***						
**ABA signaling/Ca signal** **transducer**	PPN027B08	Calcium-dependent proteinkinase	CPK32	CS-glob1	S>LS	ABA, salinity
	PPN029E04	GTP-binding protein-related.	MIRO2/ATCBG	CS-glob1	N/A	ABA, salinity
	PPN031C02	Rac-GTP binding protein-like	MIRO2/ATCBG	CS-glob2	S>LS	ABA, salinity
	PPN069F09	PK11-C1	OST1//SRK2E	CS-glob6	N/A	ABA, osmotic stress
**ABA signaling/Casein kinase** **regulation**	PPN057C06	casein kinase 1 protein family	CKL2	CS-glob1	S>LS	ABA regulation
**ABA signaling/signal transducer**	PPN021G09	Protein kinase	SNF1/SRK2I	CS-glob6	S>LS**^b^**	ABA, osmotic stress
**Aux signaling/Aux receptor E3** **ubiquitin ligase SFC-TIR**	PPN070C07	F-box containing protein TIR1	AFB5	CS-glob1	S>LS	AUX signaling
	PPN078E01	TRANSPORT INHIBITOR RESPONSE 1protein	TIR1	CS-glob6	N/A	AUX signaling
**Aux signaling/pin phosphorylation**	PPN014G07	Serine/threonine-proteinphosphatase 2A reg. sub. Abeta	PDF1/PP2AA2	CS-glob6	N/A	AUX signaling
**Aux signaling/Ubiquitin ligation E3** **complex/F-box**	PPN026G02	Auxin-responsive factor TIR1-likeprotein	AFB2	CS-glob1	S>LS	AUX signaling
**Calcium signaling/Calcium** **signal transducer**	PPN011E06	CBL-interacting serine/threonine-protein kinase 11	ATSR1/CIPK14	CS-glob2	S>LS	cold, salinity and ABA
	PPN013H01	Serine/threonine kinase	CIPK10/SIP1	CS-glob11	S>LS	cold, salinity and ABA
	PPN017F05	CBL-interacting serine/threonine-protein kinase 11	CIPK11/SIP4	CS-glob6	N/A	cold, salinity and ABA
	PPN080C05	Protein kinase; NAF	CIPK1	CS-glob6	S>LS^a^	ABA, osmotic stress
**Cyclic nucleotide signaling/(p)ppGpp-** **mediated response**	PPN046D08	RelA/spoT-like protein RSH2	RSH2	CS-glob6	N/A**^b^**	ABA, salinity, wounding
**Ethylene signaling/SCF(EBF1) E3** **ubiquitin ligase**	PP1005A04	Leucine Rich Repeat, putative	EBF1	CS-glob1	N/A	ET, cold
	PPN023E11	EIN3-binding F-box protein 1	EBF2	CS-glob5	S>LS	ET, cold
**Ethylene signaling/ethylene receptor**	PPN054G06	Ethylene receptor		CS-glob2	S>LS	ET
	PPN057C10	Ethylene signaling protein	EIN2	CS-glob1	N/A	ABA, ET, cold, abioticstress
	PPN079H05	Ethylene signaling protein	EIN2	CS-glob1	N/A	ABA, ET, cold, abioticstress
**G-protein coupled receptor protein** **signaling pathway/G-protein complex**	PPN005H05	Extra-large G-protein	XLG1	CS-glob1	N/A	osmotic stress, ABA
	PPN029C06	Extra-large G-protein	XLG1	CS-glob1	S>LS	osmotic stress, ABA
	PPN065B10	Extra-large G-protein	XLG3	CS-glob6	S>LS	osmotic stress, ABA
**Light signaling/light receptor**	PPN005E08	Cryptochrome 2A apoprotein	CRY2	CS-glob3	S>LS	Light, low temperature
**Light signaling/light transducer**	PPN023G10	phototropic-responsive NPH3 family protein		CS-glob6	S>LS	light
**Phosphorylation cascades/PP2A**	PPN037E11	Serine/threonine proteinphosphatase 2A reg. sub B′gamma	ATB′GAMMA	CS-glob1	S>LS	light, defense response
**Phosphorylation cascades/PP2C**	PP1005B01	protein phosphatase 2C,putative	PP2CG1	CS-glob6	S>LS	ABA, drought, salinity
***Intracellular traffiking***						
**ER to Golgi**	PP1003D05	Root hair defective 3	RHD3	CS-glob5	LS>S	AUX, ET
**ESCRT-dependent protein sorting** **and concentration**	PPN005D10	Putative vacuolar sorting protein 35	VPS35A	CS-glob5	LS>S	AUX transport regulation
	PPN026H03	Putative vacuolar sorting protein 35	VPS35A	CS-glob1	LS>S	AUX transport regulation
***Transport***						
**Nucleocytoplasmic transport**	PPN023D05	Peptidase S59, nucleoporin	SAR3/MOS3	CS-glob1	N/A	AUX-regulated nuclear transport
**Trans-Golgi network transport** **vesicle/COPI vesicles**	PPN002C04	ARF-GAP	SFC	CS-glob5	LS>S	AUX transportregulation
**Aux transport**	PP1004E09	auxin efflux carrier family protein		CS-glob8	LS>S^b^	AUX
	PPN058C04	Auxin efflux carrier protein-like		CS-glob6	LS>S	AUX
	PPN075H08	auxin efflux carrier family protein		CS-glob8	LS>S	AUX
**Fe-S cluster maintenance and response** **to far red light**	PPN024F02	Protein NAP1, chloroplastprecursor	NAP/LAF6	CS-glob3	S>LS	Light
**Lead tolerance**	PPN032F06	PDR-like ABC-transporter	PDR12	CS-glob1	LS>S^a^	ABA, drought
**Na/K antiporter**	PPN064A01	Na+/H+ antiporter	SOS1	CS-glob1	LS>S	salinity, ion homeostasis

a contribution to PC2 (Fig. 1A) positive; b positive correlation with projected MI.

To see references supporting the involvement of these genes in stress and/or hormones see Table S3.

In order to analyze if the transcript program in the cold may have a direct effect on eventual mealiness development during shelf life, a Pearson correlation analysis was conducted between the gene expression values and the projected MI will be achieved when subjected to shelf life ripening after cold exposure (the actual MI of cold stored samples were 0 as shelf ripening is required to develop mealiness). This “projected MI” correlation analysis resulted in 113 directly correlated genes (R>0.8) and 159 inversely correlated genes (R>0.8) according to their pattern of expression in the cold (Fig. S3 in File S1 and Table S2). The functional enrichment analysis (Fig. 2C and Results S3) indicated that genes directly correlated to projected MI were enriched in *RNA transcription* and *RNA posttranscriptional regulation.* A further inspection revealed genes related to *RNA biogenesis and processing, splicing, RNA transcription machinery* and the *transcription factors* (Table S3). In addition, genes correlated positively with the projected MI were also enriched in *transport* category (Fig. 2C, Results S3), that includes transporters for auxin, anthocyanin, amino acid, peptides, sulfate, carbohydrates and metal-ions (see Table S3). No functional enrichment was observed for those genes which correlated negatively with projected MI (Fig. 2D). However, a detailed inspection indicated that this set of genes contained calcium-related genes, including a transcription factor of the CAMTA family, and genes related to antioxidant systems (Table S3) which could participate in the regulation of this transient tolerance mechanism.

### Is there a Preprogrammed Mechanism for Chilling Tolerance?

**Figure 3:**
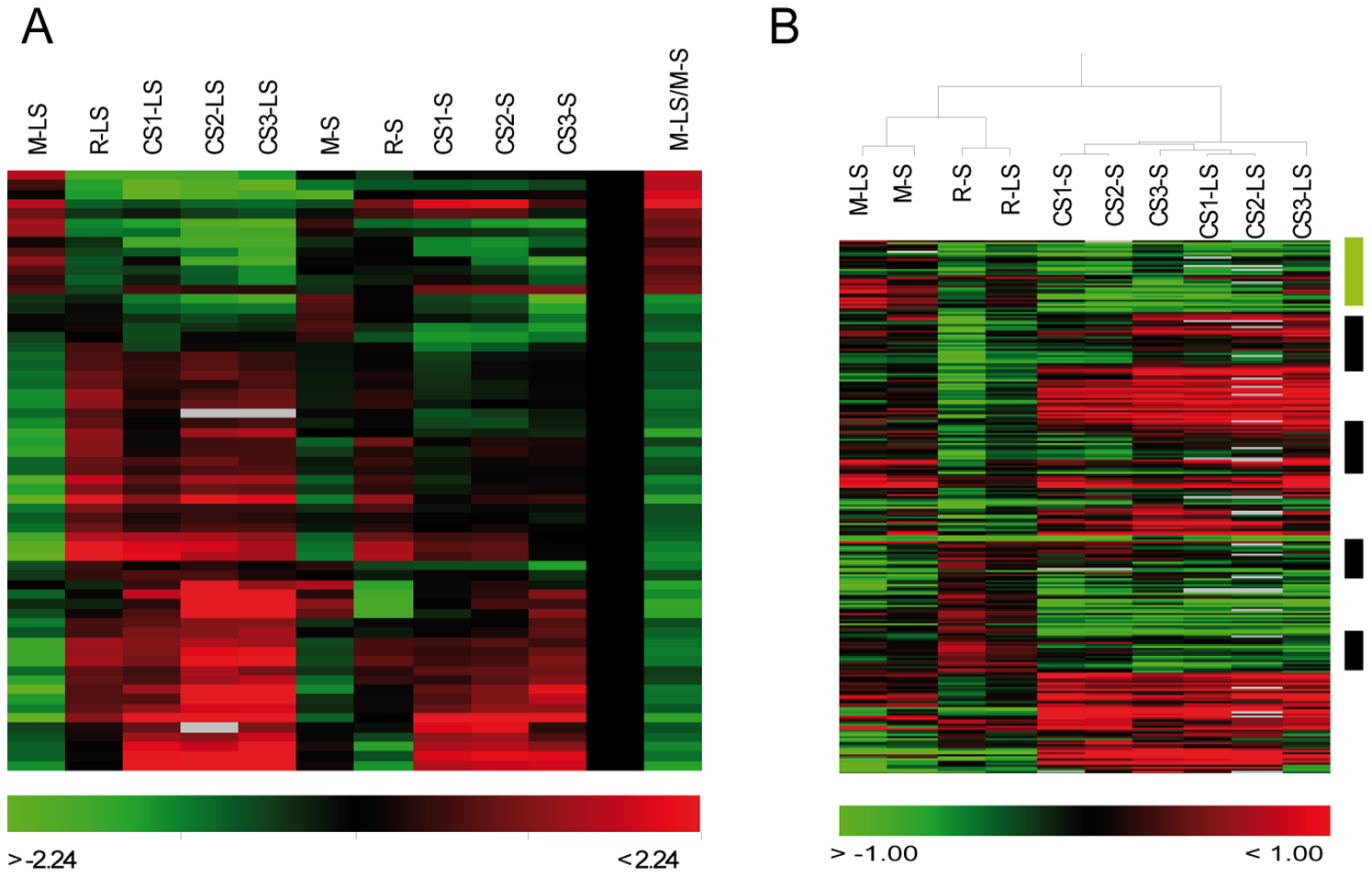
Preformed mechanisms and effect of ripening. A) The hierarchical cluster of the 63 genes differentially expressed between fruits LS and S at the mature stage. The expression values for samples M, R and CS and the M-LS *vs.* M-S ratio are shown. B) Hierarchical clustering of the expression values for 862 ripening genes (up or down in fruits R respect to M) during cold storage. M = mature fruits, R = mature with 2–4 days ripening at 20°C, CS1 = M +1 week cold storage at 5°C, CS2 = M +2 weeks cold storage at 5°C, CS3 = M +3 weeks cold storage at 5°C.

The possibility that, in addition to cold-inducible mechanisms, some sort of tolerance mechanism may already be partly preprogrammed in tolerant fruits was investigated. The direct comparison between S and LS fruits at mature stage (M) resulted in 63 differentially expressed genes (Fig. 3A and Table S3). Out of them, 13 genes we high expressed in fruits T (Table S3) and some have to do with flavonoid metabolism (CHS/TT4 and GST12/TT19), structure protection (Tic110) and (ASN1/DIN6) that forms part of a cycle that generates asparagine for more energy-economical nitrogen remobilization under darkness and stress conditions [64], [65]. Several cell wall modifying activities were also differentially expressed between fruits S and LS (Table S3). As no differences at the maturity stage were between pools (Table S1), it is likely that differences in the expression levels of these genes at harvest may protect fruits and/or contribute to develop the tolerance program at least in the early stages of the cold response.

HCA of samples M, R and CS (Fig. 3A) showed that genes differentially expressed between fruits S and LS at harvest qualified in fruits LS as ripening genes (see columns 1 and 2 column in the cluster; Fig. 3A). Notwithstanding, it is most interesting to note these genes were characterized by continuing the ripening program during cold storage (see the CS-LS samples and compare with R-LS), which did not happen so clearly in fruits S (compare the CS-S samples and compare with R-S). However and as expected this behavior of the differential M genes is the exception rather than the rule for ripening genes. As seen in Figure 3B, a similar analysis with a set of 862 ripening genes (up or down regulated in R by comparing to M) showed that although cold affect the expression many of ripening genes, is quite effective stopping the molecular ripening program in fruits LS. This result is in agreement with the findings from PC2 (see Fig. 1B). The main expression differences between LS and S fruits involved changes occurring in the same direction in R and cold stored fruits. In fruits LS, the expression of several ripening genes during cold storage remained at the same or higher level that they were in the M stage, but achieved similar expression levels to fruits R in the sensitive backgrounds (black bars in Fig. 3B). Apart from the delayed or attenuated ripening program in the fruits LS during cold storage, these fruits also showed specific ripening processes that became activated during cold storage (green bar in Fig. 3B), which is in agreement with the findings for genes differentially expressed at harvest (Fig. 3A). A more detailed analysis of shelf life ripening conditions and mealiness will be addressed in a future manuscript (in preparation).

### Cold Regulons in Peach Contributing to the Differential Response to Cold

In this section we wanted to see if there were similarities between the adaptation mechanisms operating in peach fruits stored in cold and darkness and those well-characterized in the cold acclimation of *Arabidopsis* plants grown in day/night regimes. We wanted to see if the patterns of gene expression for the peach homologues of Arabidopsis genes in cold/dehydration regulons were consistent with the differential cold responses in S and LS peaches.

**Figure 4:**
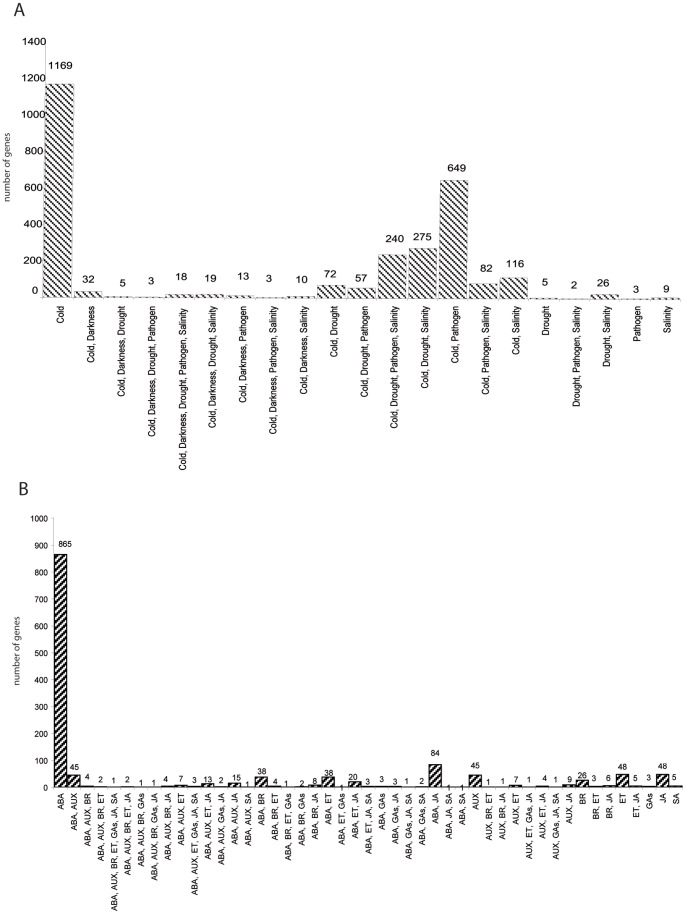
Comparison of the chillpeach data with the available microarray public domain data. A) The differentially expressed peach genes in the global analysis (Fig. 1) and reported as cold and/or Stress Response genes. B) The differentially expressed peach genes in the global analysis (Fig. 1) and reported as hormone responsive genes.

First we analyzed the overlap between the response of cold stored peach fruits and those to various stimuli, including abiotic/biotic stresses and hormones (Method S1 and Fig. 4). Gene-by-gene comparisons revealed that the vast majority of the cold-regulated genes in our peach cold storage experiment have *Arabidopsis* orthologs, which have been described as being regulated by cold (63%, Fig. 4A), or by ABA (35%, Fig. 4B). Similarly to *Arabidopsis* [66], approximately 30% of peach cold-regulated genes were found to be associated with drought and/or salinity treatments (Fig. 4A). More strikingly however, approximately 35% of the cold-responsive genes in peach were known pathogen-responsive genes or have been postulated to play a role in pathogen resistance (Fig. 4A). Furthermore, the genes described as being regulated by darkness in *Arabidopsis* account for up to 3.7% of peach cold-regulated genes (Fig. 4A), indicating that, although its contribution to all cold-regulated genes was less than those also involved in other stresses, dark stress could contribute to the differences observed in the cold response between peach fruits (dark) and *Arabidopsis* plants (light).

Second, a list of *Arabidopsis* genes reported in cold regulons (CBF, ZAT12, HOS9, HOS15 and GI) and dehydration regulons (ESK1, AREB/ABF, MYC- DREB2, ZF-HD/NAC and CBF4) (see Table S5 and references within) was used to identify homologous peach genes that were present on Chillpeach microarray (see Table S6). In total, 163 Chillpeach unigenes corresponded to the genes found in at least in one of the previously defined cold and/or dehydration *Arabidopsis* regulons (Table S6). The expression profiles of these genes in response to cold storage were compared to those described for *Arabidopsis* (related either to non treated plants or cold-sensitive *Arab* mutants, or both) and scored as matching when they behave similarly. More than 60% of the genes associated to the regulons CBF, HOS9, ICE and DREB2 correlated well with both the known *Arabidopsis* WT cold response pattern and the *Arabidopsis* mutant expression (Table 4). That is, the ortologs genes to those up-regulated in *Arabidopsis* in response to cold showed higher expression levels in LS peach fruits than in high sensitive ones, while the genes down-regulated in *Arabidopsis* had higher levels in high sensitive peach fruits than in low sensitive ones. In contrast, most of the genes in HOS15, ZAT12, ESK, AREB, MYB, ZF/HD-NAC presented low correlation levels (Table 4). Therefore, these latter are less likely to contribute to the differences in response to cold between the S and LS pools of fruits.

**Table 4:** Cold Regulons in Peach Fruits contributing to the Differential Response to Cold Storage.

		Data for genes in each regulon and percentage of correlation			PCA					Hierarchical cluster		Importance of regulon	
DATASETS	Regulon	Genes inArabidopsis	Genes found in chillpeach	%of geneswellcorrelated	PC1	PC2	PC3	PC1 separateCS1-LS fromCS1-S	PC2 separateCS1-LS fromCS1-S	Weight of thenearest node to CS1-LS	CS1-LS branchedout of CS samples	To discriminateS from LSsamples	To separate samples that will become mealy or not
**Cold and** **dehydration** **regulons**	**all**	1236	163		64.9	14.0	9.5	•	√		√		
**Cold**	**CBF**	187	31	74.19	65.0	15.0	9.0	•	√√	0.69	√	3.45	33.33
**Cold**	**HOS9**	154	14	78.57	70.5	18.4	5.2	√	√√	0.142	√	2.024	77.46
**Cold**	**HOS15**	135	10	40.00	60.0	20.0	10.0	•	•	0.85	•	0.8	4.71
**Cold**	**ICE1**	369	46	60.87	55.0	24.0	10.0	•	√√	0.31	√	6.72	90.32
**Cold/** **Dehydration**	**ESK1**	310	42	47.62	75.0	11.0	6.0	•	•	0.671	•	2.2	29.81
**ABA** **DEPENDENT**	**AREB**	99	17	52.94	66.0	21.0	5.0	•	√√	0.696	√	1.89	12.93
**ABA** **DEPENDENT**	**MYC-MYB**	35	8	37.50	66.0	22.0	7.0	•	√	0.942	•	0.66	3.18
**ABA** **INDEPENDENT**	**DREB2**	45	10	70.00	70.0	16.0	7.0	•	•	0.601	√	1.12	11.65
**ABA** **INDEPENDENT**	**ZF-HD/NAC**	83	17	35.29	67.0	18.0	7.0	•	√	0.934	•	1.08	6.42
**Cold**	**ZAT12**	26	3	33.33									
**Cold**	**GIGANTEA**	1	1	100.00									
**ABA** **DEPENDENT**	**CBF4**	78	1	0.00									

√ the property analyzed is fulfilled.

• the property isn’t fulfilled.

√√ indicates that the property is fulfilled but there is a high degree of separation between samples CS1-LS and CS1-S.

The table shows the number of members of each regulon described for Arabidopsis, the number of genes found in Chillpeach, the number of genes whose expression correlated with those described for the Arabidopsis WT, PCA and 2DHCA properties. The importance of each regulón based on both the variance explained by component 2 of the PCA and the weight of the nearest node to CS1-LS. For each dataset, it is indicated if the genes in the dataset fulfill the PCA and cluster properties or not.

The individual participation of each regulon to the differential response to cold between fruits S and LS was assessed by studying their contribution to the traits/trends observed in the global dataset analysis. For this purpose, we performed both PCA and 2D-HCA (Fig. S4 in File S1) using the gene expression values for all the genes in each regulon as input datasets and quantitatively evaluate the importance of each regulon (i) to discriminate samples S from LS and (ii) to separate the samples that would eventually became mealy, or not, by assessing by the number of genes well correlated with *Arabidopsis* in the gene expression models (the PCA and 2D-HCA in Fig. S4 in File S1). The importance to discriminate samples S from samples LS (Table 4) was calculated by multiplying the number of genes that correlated well by the variance explained by PC2. The importance of an operon to separate the samples that would eventually become mealy, or not (Table 4), was quantified by dividing the number of genes in that operon that correlated well by the weight of the nearest node to CS1-LS. Both PCA and 2D-HCA revealed that regulon ICE1 was the one most contributing to discriminate samples LS and S, as to separate samples CS1-LS from the rest of cold-stored fruits that developed mealiness when submitted to shelf life ripening (Table 4). Furthermore, this analysis also indicated that the regulon CBF1 was the next major regulon in discriminating between samples LS and S (Table 4), while emphasized the relevance of HOS9 to separate CS1-LS from the remaining samples (Table 4). The rest of the cold operons produced no such separation between CS1 S and LS, or did so but to a lesser extent (Table 4 and. Fig. S4 in File S1). The expression pattern of the subsets the genes appertaining to the regulons ICE1 (46 genes), CBF (31 genes) and HOS9 (13 genes) across the different samples (Fig. S5 in File S1) showed that although extended exposure to cold debilitated the response of ICE-CBF regulated genes, fruits LS were able to maintain a longer and greater response for many of the genes in the(se) regulon(s) in the cold. In the case of HOS9 regulon, many of its members were up-regulated or without change in LS fruits as compared to M fruits (Fig. S5C in File S1).

### Validation and Extension of Microarray Expression Profiling

**Figure 5:**
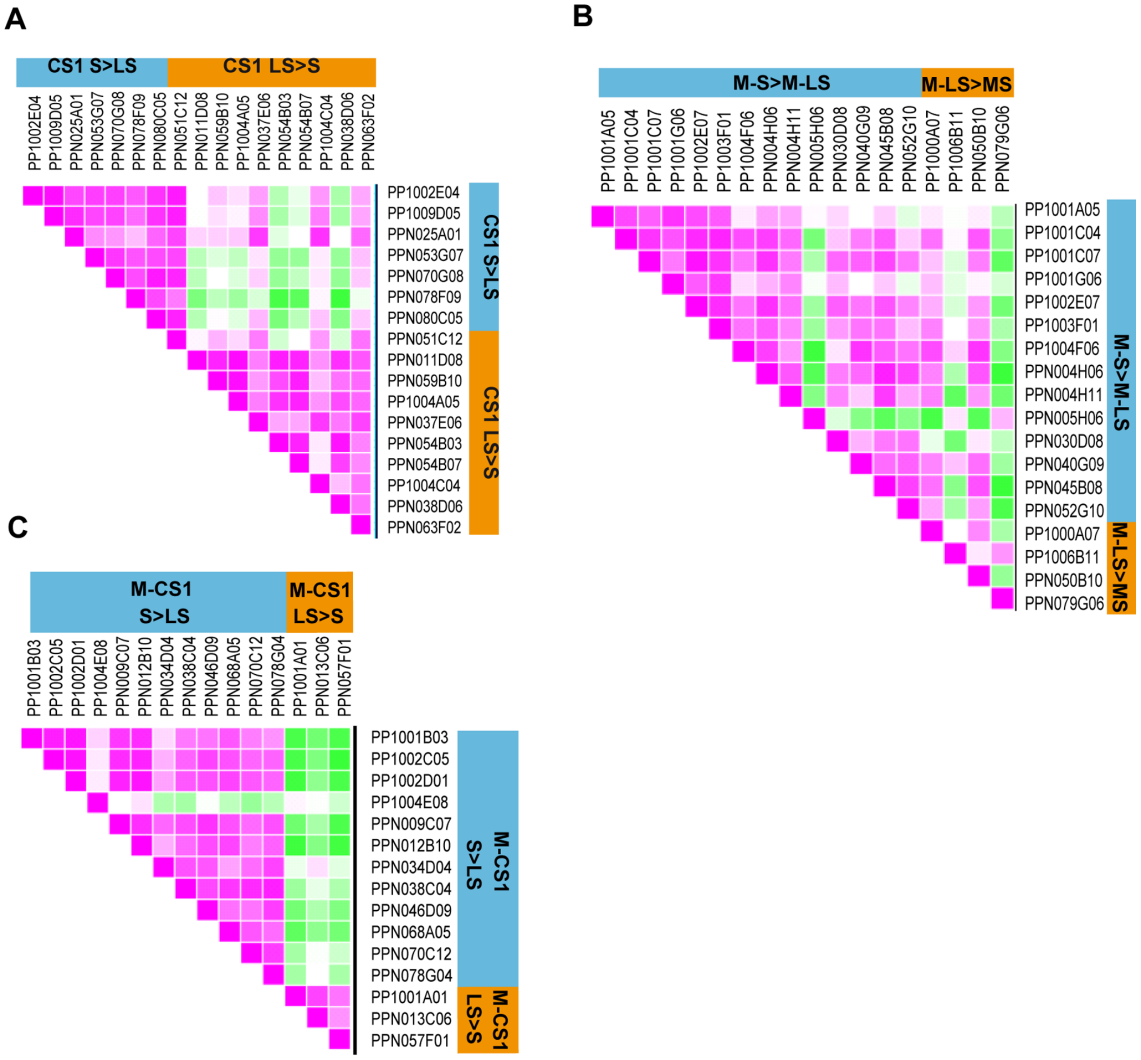
Degree of association between the genes validated by Fluidigm in a pre-defined expression pattern from the pools in the microarray and in individual Pop-DG siblings. A) The differentially expressed genes at 1 week of cold storage; B) The differentially expressed genes in the M stage and at 1 week of cold storage; C) The differentially expressed genes in the M stage. The Heatmap values correspond to the Pearson correlation coefficients between pairs of genes. For each gene in a gene set, the expression profile from the microarray results was defined and the Pearson correlation coefficients were calculated for pairs of genes in the individual sibling lines.

The same bulked samples used in this microarray experiment were used to validate the results by using medium-throughput qRT-PCR (Biomark Dynamic Array, Fluidigm) over a set of genes selected because they 1) contributed to separate samples S from samples LS at 1 week of cold storage (Fig. 1A and 2A), 2) showed a differential expression in, both, the M stage and 1-week of cold storage (Fig. 2A and 3A), and 3) showed differences at harvest (candidates to the preprogrammed mechanism of tolerance; Fig. 3A). In order to examine at the single sibling level the reliability of the differential gene expression patterns obtained from the pools, the analysis was performed also on 15 individual genotypes of the pop-DG population (those used in the pools and others showing differences in mealiness phenotype). The qRT-PCR results obtained from the pools and from the individual lines making up this pools indicate that 72.5% (50 of 69) of the genes had the same expression pattern in the microarray experiment as in the qRT-PCR experiment (Table S8). However, the magnitude of expression varied slightly in many of the genes and samples tested (Fig. S6 in File S1). Furthermore qRT-PCR experiments conducted on individual pop-DG siblings revealed that 42 out of the 50 genes validated in the pools were consistent with the expected patterns for which they were selected (Fig. 5). These results support the validity of our approach and indicate that the genes selected from the microarray analysis could be either involved in chilling tolerance and/or be associated with the differential response to chilling response, and for some of them could even prove to general enough to hold true in individual fruits/plants.

## Discussion

Since cold induced mealiness is not observed until the cold stored fruit are allowed to ripen, the chilling sensitivity phenotype of each fruit in the cold was estimated from the protracted mealiness incidence observed for equivalent fruit samples after shelf life ripening (Fig. 1A). Although mealiness, probably, a downstream effect of cold stress in peach fruits (as is also the case for the growth retardation of the electrolyte leakage used to measure the effect of cold in vegetative tissues such as *Arabiodpsis*), it is the best phenotyping tool to assess the effect of cold on peach fruit, and has be used successfully to identify CI QTLs in peach [33], [35].

For BSGA we use Chillpeach microarray [45], interrogating part of peach genome. This provides only an incomplete picture of the genes behind the process; that is partially compensated by Chillpeach microarray being enriched in fruit-specific and cold responsive genes [45].

Our study differs from prior peach transcriptomic analyses in two ways. First, we are using samples from pools of genetically related siblings with contrasting sensitivity to chilling injury subjected or not to cold storage. Thus we expect to reveal genes whose expression patterns are linked to the different cold sensitivity, while leveraging transcript differences associated with other phenotypic traits, as it would be the case when comparing only two peach cultivars that have different chilling susceptibilities in addition to other phenotypic differences. Second, by medium-throughput qRT PCR we extended our microarray results derived from the comparison of the contrasting pools to a relatively large number of 15 individual lines from the same population differing in the mealiness sensibility and the gene expression results of the selected genes were consistent with their individual sensitivity level.

### Cold Storage Conditions Induces an Acclimation Program in Peach Fruits Only to be more Effective in Tolerant than in Sensitive Fruits

Orthologs of several transcription factors (TF) found up-regulated similarly in S and LS cold-treated fruits (Table 1) have been previously reported as being up-regulated during cold acclimation in *Arabidopsis* (see Table S4 for references) and some of them also were described as belonging to a given cold acclimation regulon [9], [20]. This suggests the activation of a cold response program in peach fruits in part similar to those described for *Arabidopsis* cold acclimation. Despite observing similarities some genes exhibited an opposite trend compared to *Arabidopsis* (Table 1) which may partially reflect the sensitive character of peach fruit to cold (both LS and S fruits are sensitive, but LS fruits are relatively more tolerant than S). Several studies have associated cold tolerance and cold acclimation the transcriptional activation of genes encoding heat-shock proteins (HSPs), chaperonins, LEA proteins, antioxidant/scavenging systems and related to protein synthesis [1], [67], [68], [69]. Genes in these functional categories were generally down-regulated by cold storage in both LS and S fruits, what correlates well with their sensitivity to cold. Further, the orthologs of HSF4B and HSP21 (Table 1) were up-regulated peach fruits, whilst were down-regulated in Arabidopsis. This is particularly interesting as these genes are highly up-regulated in *Arabidopsis* chilling sensitive mutants upon chilling treatment [69], [9]. It should be noted that we are comparing the transcriptomes of different species and tissues at various physiological and growth stages, and it is likely that some differences in strategies (efficient or not) to cope with exposure to low temperatures operate in each case [70]. The basic question is: why do LS Pop-GG siblings tolerate better cold storage than S? Our results indicate that during cold storage fruits LS maintain higher levels of expression for a series of components of the antioxidant system, structure maintenance proteins and protein synthesis at least during the first week of storage (Fig. 1D, 2B and Table S3). In addition, the orthologs of some TF with a higher expression levels in tolerant peach fruits (Table 2) have been reported to be up-regulated by cold and/or other biotic or abiotic stresses in Arabidopsis (Table S4). All this supports the idea of the existence of an acclimation program more effective in fruits LS. In this sense, our data indicated that the peach orthologs for genes in ICE1, CBF and HOS9 regulons may be implicated in the tolerance of fruits LS. The central role played by the ICE1-CBF cold response pathway in cold acclimation and cold tolerance is well-established in plants [71] and has been demonstrated to exist in a wide range of plants [72], [73], [74], although, there are differences in the regulation or the size of their CBF regulons [26], [27]. The existence of ICE-CBF pathway has been also confirmed in fruits [73], [74]. Further, LeCBF1 expression levels correlates positively with cold tolerance in tomato fruits [25]. We found that genes in the regulons ICE1and CBF were the most contributing to discriminate samples S from LS, and/or to separate samples that will become mealy, or not (Table 2 and 4). Moreover, PCA analysis identified CBF1 as the second gene that contribute the most to separate the S and LS series (Fig. 1B and Table S3) and qRT PCR analysis showed that the expression levels of CBF1(PPN054B03) correlate well with the tolerance/sensitivity of the individual pop-DG siblings (Fig. 5). Thus, confirming ICE-CBF as important actors in the differential response to chilling between peaches S and LS. In the case of the genes in regulon HOS9 our results suggest that it is more likely related with the ability to up-regulate or to maintain similar expression levels to those observed in M fruits (Fig. S4C). Zhu et al. [10] concluded that HOS9 must be important for both the constitutive expression and cold-induced expression of the genes that may be required for full tolerance to freezing stress. These results are consistent with peaches having the basic components of a cold response pathway, but additional studies will be required to elucidate their size and how they are regulated.

In normal commercial fruit operations cold storage, involves also complete darkness. Gene by gene comparisons has revealed that around 3% of our cold regulated genes in peaches could be related to darkness (Fig. 4A). Moreover, we identify some genes whose orthologs have been described in the regulation or in response to light (Table 1, 2 3 and Table S4). Several, light sinaling elements among which were GI [75], DFL2 [76], PHYA [77] and FYPP3 [78] were repressed by cold storage in both LS and S (Table S3), consistently with the storage in darkness conditions. In addition, genes differentially expressed between fruits S and T include a number of regulators involved in light response (Tables 2 and 3 and for references Table S4) that indicates we should take into account this factor as contributing the differential response observed in peach fruits. In *Arabidopsis* light is required for cold induction of several genes involved in cold acclimation, including CBF*s* [79], [80] and some light signaling mutants have impaired cold acclimation [81]. Thus the differential response to cold storage of fruits S and LS probably have to do fruits’ ability to deal with cold and darkness. However, further experiments are required to determine in more detail the nature of the interaction between the cold and the darkness during storage.

### Altered and Continued Ripening Associated Dehydration/Osmotic Stress could be Related with the Sensitivity of Peach Fruits to Cold

Despite no visible mealiness symptoms are observed during cold storage, the BSGA indicated dramatic changes in the peach transcriptome in response to the exposure to mealiness-inducing temperatures in a manner that these changes could be useful to predict future mealiness development (Fig. 1B, 2 and S3 in File S1). We propose the transcript differences observed while in the cold might underlie the molecular basis of a mealiness phenotype which is still undetectable, but will be fully developed later during shelf life. This is in agreement with previous reports of the cold induction of specific target genes that are associated with the mealiness disorder [41], [43]. Surprisingly, our results showed that cell wall is not found among enriched categories in none of the clusters/comparisons performed on cold stored samples, suggesting that although specific changes in cell wall remodeling transcript are detected (Table S3) most of the changes would probably occur during shelf life [39].

Our results reveal also that *transport* and *signaling elements* (Fig. 2B) presented higher levels in S fruits, which in some cases, correlated well with the eventual mealiness phenotype. We found the orthologs of genes described as positive regulators of ABA signaling and/or osmotic stress (Tables 3 and S4) and transporters related to Na+ and K+, sugar and nitrate homeostasis (Table S3) among genes high expressed in fruits S This suggests that fruits S during cold storage undergo some sort of dehydration or osmotic adjustment. It has been proposed that during cold storage, before mealiness is manifested, pectin depolymerisation but not de-esterification is inhibited [37], [38], [39], what may lead to the formation of gel-forming pectins that traps free water from the surrounding tissue. As no significant differences in global water content are found between LS and S fruits (A.Dagar, personal communication) it is likely that water is being lost from the cell to be trapped on the pectins of the cell wall, which still would be sensed as loss of internal water by the cell.

Among genes with higher expression in sensitive fruits we identified components of auxin and ethylene signaling cascades as well the orthologs of genes involved in the biosynthesis of ABA, auxin and ethylene (Tables 3 and S4). We must highlight the large list of genes related to auxins among with were positive regulators of auxin responses and transporter locations (Table 3 and Table S4). In addition, among the genes high expressed in the fruits LS at one week (Table 2) there were the orthologs of genes such as HAB1 [82], PP2CA/AHG3 [83], SAD1 [84] and ERD15 [85], which have all been described as negative regulators of ABA signaling, and IAA17/AUX3, proposed to be a negative regulator in auxin and ABA signaling [86]. Ethylene and auxins has been described in the regulation of the ripening program of peach fruits [87] and their involvement in the cold response has been described for Arabidopsis [20], [23], tomato [88], apple [28] and peach [89]. Our results indicate that part of the ripening program probably continues during cold storage in sensitive fruits (Fig. 1B and 3B). Hence, we could expect that interactions between cold and hormones controlling the peach ripening program, which are differential between fruits S and T, impact the way fruits respond to cold and ripen afterwards during shelf life. Because the activity of most of these genes is mainly determined at post-trasncriptional level reviewed in [90], it is not possible from expression data only to infer the role of these genes during cold storage. However from our data it is clear that all three hormones may play a role in regulating the differential response of peach fruits to cold and they seem operate in association with dehydration/osmotic stress. In support of that, the orthologs of many of hormone related genes higher expressed in CS1-S fruits have been described previously either in relation to drought and osmotic stress (Table 3). For example, the orthologs of SKIP [91], BRM [92] and ERD1 [93], [94]mediate the responses or are induced by ABA, salinity and dehydration stress; CPL2 modulates auxin responses, plant growth and osmotic (salinity) stress [95] and EIN2 has been described to be an important cross-link node for the interaction of ethylene, ABA and plant response to abiotic stress [96].

We cannot rule out that the “sensitivity” program is the consequence or the cause of low levels ICE1-CBF regulons. It is possible that the up-regulation of a set of common genes (cluster CS-glob8, Fig. 1C) concomitantly with low CBF levels triggers this program. It is also feasible that among CS1 S>LS there are genes which negatively regulate the CBF response. To support this, EIN2 (Table 3) has been described as a negative regulator of plant response to freezing stress by negatively regulating the expression of CBF1-3 and its target genes [23]; interestingly, CBF genes have been found to be directly repressed by IAA [97]. Finally, it may also be possible that this program is activated to compensate efficient acclimation during cold storage. It has been described that *hos9* mutants hyperactivate some cold-regulated genes through a compensating response to their increased cold sensitivity [10].

### A Preprogrammed Mechanism Contributes to Chilling Tolerance

At the mature stage specific differences at the gene expression level between the pools of fruits S and T already exist (Fig. 3A). Although our approach used pools of fruits in accordance to how they respond to cold storage, therefore minimizing differences in other aspects between genotypes, we can’t dismiss the possibility that these differences have nothing to do with adaptation to cold. Preformed mechanisms have been described in both biotic and abiotic stress tolerance [98], [99], [100] and we previously identified a subset of genes differentially expressed at harvest that correlate well with CI [46].

Cell wall metabolism has been extensively related to mealiness in peach fruits [37], [38], [39], and it has been reported that endo-polygalacturonase plays a qualitative role in the mealiness expression [33]. Our results indicate that the composition of the cell wall at harvest could play a role in the tolerance or sensitivity of peach fruits to withstand cold storage. This is in agreement with previous results [46]. In addition the type of functional categories for the differentially expressed genes at the stage M, and the fact that most of these genes continue to show these differences during cold storage (Fig. 3A and Table S3), suggest the possibility that a pre-programmed tolerance/sensitivity mechanism can be partly established previously to cold. Among the highly expressed genes in fruits LS at the mature stage, we found orthologs of genes such as CHS/TT4 and GST12/TT19 (Table S3), which have been described being essential for anthocyanin and proanthocyanin accumulation [101], [102]. Anthocyanins have been related with browning in peaches [34]. However, no significant differences in browning, bleeding (Table S1) nor in ppLDOX expression (Table S3) were observed between our pools. It is suggested that AtTT19 functions as a carrier to transport proanthocyanin precursors to the tonoplast [103] to be later secreted and linked to cell wall polysaccharides [104]. Binding that depends on the composition of the proanthocyanin [105]. The tt19 mutation leads to the formation of aberrant PA derivatives [103]. Thus is possible that differences in TT19 have to do with cell wall composition and chilling sensitivity. Further experiments are required to test this hypothesis.

In addition, flavonoids act as negative regulators of auxin transport [101]. It is noteworthy that at harvest only two transcription factors (PAP2/IAA27 and IAA16) were differentially expressed, both showing higher expressions in T fruits and in the case of the ortholog of PAP2/IAA27, also at 1 week of cold storage (Table 2). SlIAA27 silencing results in greater auxin sensitivity in tomato [106]. Moreover, a gain-of-function mutation in IAA16 confers poorer responses to auxins and ABA in *Arabidopsis* [107]. Thus, it is likely that high levels of these genes at harvest contribute to delay the ripening program or protect fruits LS during cold storage, at least at the beginning of cold storage.

The analysis of the expression profiles during cold of the genes differentially expressed in M fruits resulted in important and unexpected expression characteristics. In fruits LS, these genes behaved like ripening genes (Fig. 3A) and were able to continue with the ripening program in the cold in fruits LS, while the ripening expression of other ripening genes was normally halted (Fig. 3B), which is not the case of high sensitive fruits. The ability of cold to stop fruit ripening has been previously reported [108], even if no details of how this happens at the molecular level have yet been provided. Although we have no hypothesis about why these genes continued with the ripening program in the cold (thus we expect that cold stopped ripening program efficiently in fruits LS), we believe that this may be because these genes are part of the adaptation mechanism or simply reflected that LS fruits perform better in the cold than S fruits. In apples the ability to set up ripening during cold seems to be an adaptative mechanism to shorten ripening time in colder autumns [28]. On the other hand, this unexpected behavior of some of the genes differentially expressed at harvest indicates that they not only can form part of a mechanism for the interaction between endogenous and exogenous signals, they could also be able to contribute to mealiness in response to cold stress. In light of this, it is interesting to remember that environmental/ripening stage/cultural preharvest practices have a strong effect on CI sensitivity during the postharvest [31], [60], [109], [110] which, together with the genetic background, may be responsible for the differences noted in the M stage that condition the cold response.

### Conclusions

In summary, using a BSGA approach we identified many new peach cold-regulated genes and discussed their possible impact on the sensitivity and tolerance in fruits. This information provides the foundation for further experiments to explore the network of gene regulation in the cold and to determine the function of cold-responsive genes in peach fruits through mutant analysis, transgenic overexpression, and other molecular or cell biological approaches. Further the set of identified genes may be used as a road map to be validated with other peach cultivars differing in sensitivity or tolerance to chilling as a first step for breeding or postharvest technology applications.

## Supporting Information

Table S1 **Fruit quality atributes mesured at harvest with flesh firmness of 12–14 lb and storage disorders measured after one week of cold storage at 5°C plus 2 days of shelf life ripening at 20°C.**
(XLS)

Table S2 **3350** **differentially expressed genes in the global analysis.** A statistical test was performed by a SAM multiclass analysis. A gene was considered significant at a FDR <0.05 and for a p-value <0.05. The table provides ID, the Lowess M Log Ratio and functional annotations.
(XLS)

Table S3 **Summary of the results of the cold response in peach.** The table indicates a) the contribution of each gene to the separation by a given principal component, b) the cluster resulting for global 2D-HCA, c) the expression pattern at harvest and during cold storage; d) the results of the correlation analysis between an average MI and the expression profiles in samples M-CS, e) functional annotations.
(XLS)

Table S4 **References supporting information in** **tables 1**, **2** **and** **3** **.**
(XLS)

Table S5 **Arabidopsis genes reported as members of the cold and dehydration regulons.** 1236 genes distributed in the regulons of CBF, ZAT12, HOS9, HOS15, GI for cold; ESK1 for cold-dehydration and AREB/ABF, MYC-MYB, DREB2, ZF-HD/NAC and CBF4 for dehydration.
(XLS)

Table S6 **Peach genes with an Arabidopsis ‘ortholog’ reported as members of the cold and dehydration regulons.** 163 peach genes were found in one of the cold and dehydration regulons, or more, and were considered to be: CBF, ZAT12, HOS9, HOS15, GI for cold; ESK1 for cold-dehydration and AREB/ABF, MYC-MYB, DREB2, ZF-HD/NAC and CBF4 for dehydration. The table indicates the expression pattern in Arabidopsis WT, as well as the mutants and the expression pattern in the M and CS1 peach samples.
(XLS)

Table S7 **The genes selected for the Fluidigm experiment.** The conditions for the gene selection, gene annotations and expression values from microarray (reason for selection) are shown along with the sequence, length and Tm for the primers used in the qRT-PCR experiments.
(XLS)

Table S8 **Chillpeach validation and extension results.** The gene ID, the Fluidigm genes selection, the M-S/M-LS chillpeach pattern, the M-S/M-LS Fluidigm pattern, the CS LS *vs.* the S chillpeach pattern, the CS LS *vs.* the S Fluidigm pattern, validation in pools and lines and the expression values of the centered, scaled and normalized obtained in Fluidigm for the pools and lines are shown.
(XLS)

Results S1 **Functional enrichment results of the 11 resulting clusters from 2D-HCA.** Results of Fisher test for overrepresented functional categories of the clusters resulting from 2D-HCA (Fig. 1C). For each Functional category in a given cluster the file includes: the number of genes in a cluster, the number of genes over all 11 clusters, the number of genes in Chillpeach and the results from fisher exact test.
(XLS)

Results S2 **Functional enrichment results of the resulting clusters from time-by-time comparisons.** Results of Fisher test for overrepresented functional categories of the clusters resulting from CS1, CS2 and CS3 direct comparisons (Fig. 2A). For each Functional category in a given cluster the file includes: the number of genes in a cluster, the number of genes over all clusters, the number of genes in Chillpeach and the results from fisher exact test.
(XLS)

Results S3 **Functional enrichment results of Projected MI correlated genes.** Results of Fisher test for overrepresented functional categories of the sets of Projected MI correlated genes (Fig. 2A). For each Functional category in a given cluster the file includes: the number of genes in a cluster, the number of genes over all clusters, the number of genes in Chillpeach and the results from fisher exact test.
(XLS)

Methods S1 **Functional annotation of Chillpeach genes: functional categories, specific process/pathways, and relation to stress and hormones.**
(PDF)

File S1 **Figures S1–S6.** Figure S1. Frequency of the individuals with a given MI Index in the Pop-DG population.; Figure S2. The global gene expression analysis of the Chillpeach transcripts in response to cold storage; Figure S3. Projected MI correlated genes; Figure S4. 2D-HCA and PCAs for the genes in regulons ICE1, CBF1, HOS9, HOS15, ESK1, MYB-MYC, DREB2, AREB and ZF-NAC; Figure S5. The peach cold operons involved in the differential response between fruits S and LS; Figure S6. The differences between the microarray and qRT-PCR results in the magnitude of the expression levels for a selected number of genes.
(PDF)
