# Supplementary material for: A Bulk Segregant Gene Expression Analysis of a Peach Population Reveals Components of the Underlying Mechanism of the Fruit Cold Response
Source: PLoS One. 2014 Mar 5;9(3):e90706. doi: 10.1371/journal.pone.0090706 (PMC3944608; doi:10.1371/journal.pone.0090706)
Supplement: Table S4 — References supporting information in tables 1 , 2 and 3 . (XLS) [file pone.0090706.s004.xls]

	Chillpeach ID	Gene description	Arabidopsis Gene Symbol	HCA pattern	CS1 S vs LS pattern	stress/hormone	Cold regulon	References
GENES COMMONLY UP-REGULATED IN S and LS FRUITS								
RNA transcription regulation								
**AP2/EREBP family**	PPN039F03	Putative dehydration-responsive element binding protein	RAP2.4	CS-glob8	N/A	CA-DR, drought, light, ethylene		[1],[2],[3],[4]
	PPN078E06	EREBP-4 like protein		CS-glob8	N/A[c]	CA-DR		[5],[3]
**AUX/IAA family**	PPN046H05	Auxin-responsive protein IAA13	IAA13	CS-glob8	N/A	AUX negative regulation		[6,7],[8]
**C2C2-CO-like Family**	PPN075B03	zinc finger (B-box type) family protein	STH2	CS-glob8	N/A	CA-DR, light		[1],[5],[3],[9]
**C2H2 Family**	PPN046D02	Zinc finger protein 4	ZFP4	CS-glob8	N/A	CA-DR		[3]
	PPN053C05	Zinc-finger protein 1	AZF2	CS-glob8	N/A	CA-UR		[10],[3],[11]
**GRF-family**	PPN044H02	14-3-3 protein 3	GRF2	CS-glob8	N/A	CA-DR		[3]
**HD-ZIP family**	PPN047H02	Homeobox-leucine zipper protein HAT22	HAT22	CS-glob8	N/A	drought, light, carbon sensing		[12],[13]
**HSP-family**	PP1002D06	Heat shock factor	HSFB1	CS-glob8	N/A	high up-regulated in Arabidopsis *chs* mutants		[14]
	PPN001A09	Heat shock factor	HSFB1	CS-glob8	N/A	high up-regulated in Arabidopsis *chs* mutants		[14]
	PPN054G07	Heat shock factor	HSFB1	CS-glob8	N/A[c]	high up-regulated in Arabidopsis *chs* mutants		[14]
	PPN055B05	Similarity to heat shock transcription factor	HSFC1	CS-glob8	N/A	CA-UR	ICE1	[15]
	PPN077H06	Heat shock transcription factor	AT-HSFA4A	CS-glob8	N/A	CA-UR, high up-regulated in *hos15* mutants	HOS15	[5],[16]
**MADS-box family**	PPN004D05	MADS box transcription factor	SVP/AGL22	CS-glob8	N/A	CA-UR		[1],[3]
	PPN058B02	MADS box transcription factor	AGL24	CS-glob8	N/A	cold up-regulated (vernalization)		[17]
**MYB-family**	PP1006F11	MYB1	ATMYB6	CS-glob8	N/A	CA-DR		[3]
**NAC-family**	PP1001F06	NAM-like protein	ATNAC2 /anac056	CS-glob8	N/A	CA-DR		[3]
	PPN054B06	No apical meristem protein-like	anac073/ SND2	CS-glob8	N/A	CA-DR		[3]
	PPN073C10	NAM-like protein	anac083/VNI2	CS-glob8	N/A	CA-DR, ABA-mediated abiotic stress		[3],[18]
**PHD-family**	PPN035F03	hydroxyproline-rich glycoprotein family protein	EDM2	CS-glob8	N/A	defense to pathogens		[19]
	PPN051C10	ABI3-interacting protein 2	AIP2	CS-glob8	N/A	CA-UR	ICE1	[15]
**TUB-family**	PPN066C05	Tub family, putative	AtTLP1	CS-glob8	N/A	CA-UR		[3]
**WRKY-family**	PPN001D05	DNA binding protein WRKY2	WRKY3	CS-glob8	N/A	CA-DR		[3]
GENES WITH HIGH EXPRESSION IN LOW SENSITIVE FRUITS								
RNA post-transcriptional regulation								
**RNA biogenesis and processing**	PPN035E09	Dehydration-induced protein ERD15	ERD15	CS-glob9	LS>S	negative regulator ABA		[20]
	PPN048C02	Sm-like protein	SAD1	CS-glob10	LS>S	negative regulator ABA		[21]
RNA transcription regulation								
**AP2/EREBP family**	PPN049D05	similar to DREB3		CS-glob8	LS>S	cold, drought, salinity		[22]
	PPN054B03	CBF1	DREB1A/CBF3	CS-glob9	LS>S[b]	CA-UR, AUX down-regulated	ICE1/CBF	[23],[24],[25],[26],[27]
**AUX/IAA family**	PP1009D02	IAA16 protein	AXR3/IAA17	CS-glob9	LS>S	negative regulator in AUX and ABA signaling		[28]
	PPN057F01	AUX/IAA protein	PAP2/IAA27	CS-glob4	LS>S	light		[29],[30]

**b-ZIP family**	PPN049B04	BZIP transcription factor bZIP68		CS-glob10	LS>S	light, cold		[31],[32]
**C2C2-CO-like Family**	PPN050G11	zinc finger (B-box type) family protein	AT4G27310	CS-glob10	LS>S	cold	AREB/ ABF	[33]
**CAMTA family**	PPN075B05	Anther ethylene-up-regulated protein ER1	SR1	CS-glob2	LS>S[d]	cold up, salinity, defense and ET		[34],[35]
**CCAAT Family**	PPN006E07	Repressor protein	NF-YB13	CS-glob10	LS>S	darkness		[36]
**HMG-family**	PPN042B12	HMG-protein	HMGB1	CS-glob2	LS>S[d]	stress		[37]
**MYB-family**	PPN041A07	myb family transcription factor	CDC5	CS-glob9	LS>S	defense responses, light, cold		[38],[31],[32]
	PPN055C11	Sucrose responsive element binding protein	ATMYBR1/ATMYB44	CS-glob10	LS>S	cold	ICE1	[15]
**PHD-family**	PPN051C09	PHD finger protein At5g26210	AL4	CS-glob3	LS>S	cold, salinity and ABA		[39]
	PPN068F05	PHD finger protein At5g26210	AL4	CS-glob10	LS>S	cold, salinity and ABA		[39]
**RNA transcription machinery**	PPN027A09	Sigma-like factor precursor	ATSIG5	CS-glob2	LS>S	light		[40]
Protein degradation								
**Proteolysis control-Signalosome**	PPN042D08	COP9 signalosome complex subunit 8	COP9	CS-glob8	LS>S	light		[41]
Signal transduction pathway								
**ABA signaling/reversible protein dephosphorylation**	PP1009B12	Protein phosphatase 2C	ATPP2CA/AHG3	CS-glob10	LS>S	negative regulator ABA		[42]
	PPN029F02	Protein phosphatase 2C (AtP2C-HA)	HAB1	CS-glob3	LS>S	negative regulator ABA		[43]
**Aux signaling/Unknown SAUR protein**	PP1001B04	expressed protein (DUF298)	AAR3	CS-glob4	LS>S[d]	AUX response regulation		[44]
	PPN015D06	auxin-responsive family protein,(SAUR)		CS-glob4	LS>S	AUX		
	PPN051E05	auxin-responsive family protein,(SAUR)		CS-glob2	LS>S	AUX		
GENES WITH HIGH EXPRESSION IN HIGH SENSITIVE FRUITS								
Energy production								
**vacuolar ATP production and cytoplasmic PH regulation**	PPN014F01	Vacuolar H+-ATPase subunit C	DET3	CS-glob5	S>LS	Light, AUX, ABA		[45],[46],[47]
Protein degradation								
**chloroplast protease**	PPN022B02	ERD1 protein, chloroplast precursor	ERD1	CS-glob1	S>LS	ABA, drought, salinity, dark induced senescence		[48],[49],[50]
**peptidase**	PPN007E05	aminopeptidase M, similar	APM1	CS-glob1	S>LS	AUX transport regulation		[51]
**Proteolysis control-Signalosome**	PPN008B05	COP9 signalosome complex subunit 2	FUS12/ ATCSN2	CS-glob1	S>LS	light		[52]
**SCF complex assembly and disassembly**	PPN068H05	Putative TIP120 protein	CAND1	CS-glob1	N/A	AUX signaling		[53]
**Ubiquitin ligase E3 complex/SFC-culin**	PPN030D09	Cullin	AXR6/ CUL1	CS-glob1	S>LS	AUX signaling regulation, light		[54],[55],[56],[57],[58]
	PPN032E01	Cullin family	CUL3	CS-glob1	N/A	ET production, light		[59],[60]
RNA transcription regulation								
**ARF-family**	PPN051B02	Auxin response factor 2	NPH4/ ARF7/TIR5	CS-glob5	N/A	AUX response regulator, cold		[61],[62],[63],[64],[15]
	PPN072B07	Auxin response factor 5	MP/ARF5	CS-glob7	S>LS	AUX signaling and transport regulator		[65],[66]
**b-HLH family**	PPN080F10	Prf interactor 30137	LHW	CS-glob6	S>LS	AUX signaling		[67]
**GRAS-family**	PPN078C08	GRAS1	SCL14/GAI/SCR	CS-glob8	S>LS	CA-UR		[1],[15],[3]
**GroTLE transcription corepressor family**	PPN076D05	Transcriptional corepressor LEUNIG	LUG	CS-glob1	S>LS	AUX signaling regulator		[68],[69]
**HB-family**	PPN069A12	BEL1-like homeodomain transcription factor	BLH1	CS-glob5	S>LS	drought, salinity		[70],[12]
**MADS-box family**	PP1009H08	MADS box transcription factor	AGL24	CS-glob8	S>LS	cold up-regulated (vernalization)		[17]
**MYB-family**	PPN058F01	GAMYB-binding protein	SKIP1	CS-glob1	S>LS	ABA, drought, salinity		[71]
**NAC-family**	PPN023B05	NAC domain-containing protein 78	NAC2/anac078	CS-glob1	S>LS	AUX, ET, salinity		[72]

	PPN062G07	NAC family protein	ATAF1	CS-glob2	S>LS	ABA, drought, salinity, pathogen	[70],[73]
**RNA transcription machinery**	PPN067A07	Elongator component	ELO1	CS-glob1	N/A	ABA, AUX	[74],[75]
	PPN070H08	C-terminal domain phosphatase-like 2	CPL2	CS-glob6	S>LS[a,c]	osmotic (salinity) stress and AUX responses	[76]
**Unknown transcription coactivator**	PPN063D04	COP1-Interacting Protein 7	CIP7	CS-glob2	S>LS	light	[77]
Secondary metabolism							
**Aux metabolism/ Aux biosynthesis**	PPN034D04	Flavin-containing monooxygenase, putative	YUC10	CS-glob8	S>LS	AUX biosynthesis	[78],[79],[80]
**Aux metabolism/ Aux conjugation**	PPN030D12	similar to Putative auxin-amidohydrolase precursor		CS-glob5	S>LS	AUX metabolism	
**Aux metabolism/Aux deconjugation**	PPN017F04	Auxin and ethylene responsive GH3-like protein	GH3.1	CS-glob1	S>LS	stress, AUX metabolism	[27]
**Carotenoid metabolism**	PP1005H08	Zeaxanthin epoxidase, chloroplast precursor	ABA1	CS-glob8	S>LS	ABA biosynthesis	[81]
**Ethylene biosynthesis**	PP1009G10	1-aminocyclopropane-1-carboxylate oxidase	EFE/ACO4	CS-glob2	S>LS	ET biosynthesis	[82]
Signal transduction pathway							
**ABA signaling/ Ca signal transducer**	PPN027B08	Calcium-dependent protein kinase	CPK32	CS-glob1	S>LS	ABA, salinity	[83]
	PPN029E04	GTP-binding protein-related, ..	MIRO2/ATCBG	CS-glob1	N/A	ABA, salinity	[84]
	PPN031C02	Rac-GTP binding protein-like	MIRO2/ATCBG	CS-glob2	S>LS	ABA, salinity	[84]
	PPN069F09	PK11-C1	OST1/ /SRK2E	CS-glob6	N/A	ABA, osmotic stress	[85],[86],[87]
**ABA signaling/Casein kinase regulation**	PPN057C06	casein kinase 1 protein family	CKL2	CS-glob1	S>LS	ABA regulation	[88]
**ABA signaling/signal transducer**	PPN021G09	Protein kinase	SNF1/SRK2I	CS-glob6	S>LS[c]	ABA, osmotic stress	[85],[86],[87]
**Aux signaling/ Aux receptor E3 ubiquitin ligase SFC-TIR**	PPN070C07	F-box containing protein TIR1	AFB5	CS-glob1	S>LS	AUX signaling	[89],[90]
	PPN078E01	TRANSPORT INHIBITOR RESPONSE 1 protein	TIR1	CS-glob6	N/A	AUX signaling	[91],[92]
**Aux signaling/pin phosphorylation**	PPN014G07	Serine/threonine-protein phosphatase 2A reg. sub. A beta	PDF1/PP2AA2	CS-glob6	N/A	AUX signaling	[93]
**Aux signaling/Ubiquitin ligation E3 complex/ F-box**	PPN026G02	Auxin-responsive factor TIR1-like protein	AFB2	CS-glob1	S>LS	AUX signaling	[94]
**Calcium signaling/Calcium signal transducer**	PPN011E06	CBL-interacting serine/threonine-protein kinase 11	ATSR1/CIPK14	CS-glob2	S>LS	cold, salinity and ABA	[95]
	PPN013H01	Serine/threonine kinase	CIPK10/ SIP1	CS-glob11	S>LS	cold, salinity and ABA	[95]
	PPN017F05	CBL-interacting serine/threonine-protein kinase 11	CIPK11/ SIP4	CS-glob6	N/A	cold, salinity and ABA	[95]
	PPN080C05	Protein kinase; NAF	CIPK1	CS-glob6	S>LS[a]	ABA, osmotic stress	[96]
**Cyclic nucleotide signaling/(p)ppGpp-mediated response**	PPN046D08	RelA/spoT-like protein RSH2	RSH2	CS-glob6	N/Ac	ABA, salinity, wounding	[97]
**Ethylene signaling/ SCF(EBF1) E3 ubiquitin ligase**	PP1005A04	Leucine Rich Repeat, putative	EBF1	CS-glob1	N/A	ET, cold	[98],[99,100]
	PPN023E11	EIN3-binding F-box protein 1	EBF2	CS-glob5	S>LS	ET, cold	[98],[99,100]
**Ethylene signaling/ethylene receptor**	PPN054G06	Ethylene receptor		CS-glob2	S>LS	ET	
	PPN057C10	Ethylene signaling protein	EIN2	CS-glob1	N/A	ABA, ET, cold, abiotic stress	[101],[102],[103],[10
	PPN079H05	Ethylene signaling protein	EIN2	CS-glob1	N/A	ABA, ET, cold, abiotic stress	4],[105],[106],[100]
**G-protein coupled receptor protein signaling pathway/G-protein complex**	PPN005H05	Extra-large G-protein	XLG1	CS-glob1	N/A	osmotic stress, ABA	[107]
	PPN029C06	Extra-large G-protein	XLG1	CS-glob1	S>LS	osmotic stress, ABA	[107]
	PPN065B10	Extra-large G-protein	XLG3	CS-glob6	S>LS	osmotic stress, ABA	[107]
**Light signaling/light receptor**	PPN005E08	Cryptochrome 2A apoprotein	CRY2	CS-glob3	S>LS	Light, low temperature	[108],[109]

**Light signaling/light transducer**	PPN023G10	phototropic-responsive NPH3 family protein	3	CS-glob6	S>LS	light	[110]
**Phosphorylation cascades/PP2A**	PPN037E11	Serine/threonine protein phosphatase 2A reg. sub B' gamma	ATB' GAMMA	CS-glob1	S>LS	light, defense response	[111]
**Phosphorylation cascades/PP2C**	PP1005B01	protein phosphatase 2C, putative	PP2CG1	CS-glob6	S>LS	ABA, drought, salinity	[112]
Trafficking machinery and membrane dynamics							
**ER to Golgi**	PP1003D05	Root hair defective 3	RHD3	CS-glob5	S>LS	AUX, ET	[113]
**ESCRT-dependent protein sorting and concentration**	PPN005D10	Putative vacuolar sorting protein 35	VPS35A	CS-glob5	S>LS	AUX transport regulation	[114]
	PPN026H03	Putative vacuolar sorting protein 35	VPS35A	CS-glob1	S>LS	AUX transport regulation	[114]
**Nucleocytoplasmic transport**	PPN023D05	Peptidase S59, nucleoporin	SAR3/ MOS3	CS-glob1	N/A	AUX-regulated nuclear transport	[115]
**Trans-Golgi network transport vesicle/ COPI vesicles**	PPN002C04	ARF-GAP	SFC	CS-glob5	S>LS	AUX transport regulation	[116]
Transport							
**Aux transport**	PP1004E09	auxin efflux carrier family protein		CS-glob8	S>T[c]	AUX	
	PPN058C04	Auxin efflux carrier protein-like		CS-glob6	S>LS	AUX	
	PPN075H08	auxin efflux carrier family protein		CS-glob8	S>LS	AUX	
**Fe-S cluster maintenance and response to far red light**	PPN024F02	Protein NAP1, chloroplast precursor	NAP/LAF6	CS-glob3	S>LS	light	[117]
**Lead tolerance**	PPN032F06	PDR-like ABC-transporter	PDR12	CS-glob1	S>LS[a]	ABA, drought	[118]
**Na/K antiporter**	PPN064A01	Na+/H+ antiporter	SOS1	CS-glob1	S>LS	salinity, ion homeostasis	[119],[120],[121]

[a] contribution to PC2 (Fig 1A) negative; [b] negative correlation with projected MI
Arabidopsis response during cold acclimation:.CA-UR cold acclimation up-regulated

Refferences

1. Kreps JA, Wu Y, Chang H-S, Zhu LS, Wang X, et al. (2002) Transcriptome Changes for Arabidopsis in Response to Salt, Osmotic, and Cold Stress. Plant Physiology 130: 2129-2141.
2. Chen W, Provart NJ, Glazebrook J, Katagiri F, Chang H-S, et al. (2002) Expression Profile Matrix of Arabidopsis Transcription Factor Genes Suggests Their Putative Functions in Response to Environmental Stresses. The Plant Cell Online 14: 559-574.
3. Hannah M, Heyer A, Hincha D (2005) A Global Survey of Gene Regulation during Cold Acclimation in Arabidopsis thaliana. PLoS Genet 1: e26.
4. Lin R-C, Park H-J, Wang H-Y (2008) Role of Arabidopsis RAP2.4 in Regulating Light- and Ethylene-Mediated Developmental Processes and Drought Stress Tolerance. Molecular Plant 1: 42-57.

5. Vogel JT, Zarka DG, Van Buskirk HA, Fowler SG, Thomashow MF (2005) Roles of the CBF2 and ZAT12 transcription factors in configuring the low temperature transcriptome of Arabidopsis. Plant J 41: 195-211.
6. Hamann LS, Benkova E, Baurle I, Kientz M, Jurgens G (2002) The Arabidopsis BODENLOS gene encodes an auxin response protein inhibiting MONOPTEROS-mediated embryo patterning. Genes Dev 16: 1610-1615.
7. Hamann LS, Mayer U, Jurgens G (1999) The auxin-insensitive bodenlos mutation affects primary root formation and apical-basal patterning in the Arabidopsis embryo. Development 126: 1387-1395.
8. Weijers D, Sauer M, Meurette O, Friml J, Ljung K, et al. (2005) Maintenance of Embryonic Auxin Distribution for Apical-Basal Patterning by PIN-FORMED–Dependent Auxin Transport in Arabidopsis. The Plant Cell Online 17: 2517-2526.
9. Datta S, Hettiarachchi C, Johansson H, Holm M (2007) SALT TOLERANCE HOMOLOG2, a B-Box Protein in Arabidopsis That Activates Transcription and Positively Regulates Light-Mediated Development. The Plant Cell Online 19: 3242-3255.
10. Sakamoto H, Maruyama K, Sakuma Y, Meshi LS, Iwabuchi M, et al. (2004) Arabidopsis Cys2/His2-Type Zinc-Finger Proteins Function as Transcription Repressors under Drought, Cold, and High-Salinity Stress Conditions. Plant Physiology 136: 2734-2746.
11. Kodaira K-S, Qin F, Tran L-SP, Maruyama K, Kidokoro S, et al. (2011) Arabidopsis Cys2/His2 Zinc-Finger Proteins AZF1 and AZF2 Negatively Regulate Abscisic Acid-Repressive and Auxin-Inducible Genes under Abiotic Stress Conditions. Plant Physiology 157: 742-756.
12. Huang D, Wu W, Abrams SR, Cutler AJ (2008) The relationship of drought-related gene expression in Arabidopsis thaliana to hormonal and environmental factors. Journal of Experimental Botany 59: 2991-3007.
13. Thum K, Shin M, Gutierrez R, Mukherjee I, Katari M, et al. (2008) An integrated genetic, genomic and systems approach defines gene networks regulated by the interaction of light and carbon signaling pathways in Arabidopsis. BMC Systems Biology 2: 31.
14. Provart NJ, Gil P, Chen W, Han B, Chang HS, et al. (2003) Gene expression phenotypes of Arabidopsis associated with sensitivity to low temperatures. Plant Physiol 132: 893-906.
15. Lee BH, Henderson DA, Zhu JK (2005) The Arabidopsis cold-responsive transcriptome and its regulation by ICE1. Plant Cell 17: 3155-3175.
16. Zhu J, Jeong JC, Zhu Y, Sokolchik I, Miyazaki S, et al. (2008) Involvement of Arabidopsis HOS15 in histone deacetylation and cold tolerance. Proceedings of the National Academy of Sciences 105: 4945-4950.
17. Michaels SD, Ditta G, Gustafson-Brown C, Pelaz S, Yanofsky M, et al. (2003) AGL24 acts as a promoter of flowering in Arabidopsis and is positively regulated by vernalization. The Plant Journal 33: 867-874.
18. Yang SD, Seo PJ, Yoon HK, Park CM (2011) The Arabidopsis NAC transcription factor VNI2 integrates abscisic acid signals into leaf senescence via the COR/RD genes. Plant Cell 23: 2155-2168.
19. Tsuchiya LS, Eulgem LS (2010) Co-option of EDM2 to distinct regulatory modules in Arabidopsis thaliana development. BMC Plant Biology 10: 203.

20. Kariola LS, Brader G, Helenius E, Li J, Heino P, et al. (2006) EARLY RESPONSIVE TO DEHYDRATION 15, a negative regulator of abscisic acid responses in Arabidopsis. Plant Physiol 142: 1559-1573.
21. Hugouvieux V, Murata Y, Young JJ, Kwak JM, Mackesy DZ, et al. (2002) Localization, ion channel regulation, and genetic interactions during abscisic acid signaling of the nuclear mRNA cap-binding protein, ABH1. Plant Physiol 130: 1276-1287.
22. Chen M, Xu Z, Xia L, Li L, Cheng X, et al. (2009) Cold-induced modulation and functional analyses of the DRE-binding transcription factor gene, GmDREB3, in soybean (Glycine max L.). Journal of Experimental Botany 60: 121-135.
23. Liu Q, Kasuga M, Sakuma Y, Abe H, Miura S, et al. (1998) Two transcription factors, DREB1 and DREB2, with an EREBP/AP2 DNA binding domain separate two cellular signal transduction pathways in drought- and low-temperature-responsive gene expression, respectively, in Arabidopsis. Plant Cell 10: 1391 - 1406.
24. Stockinger EJ, Gilmour SJ, Thomashow MF (1997) Arabidopsis thaliana CBF1 encodes an AP2 domain-containing transcriptional activator that binds to the C-repeat/DRE, a cis-acting DNA regulatory element that stimulates transcription in response to low temperature and water deficit. Proc Natl Acad Sci U S A 94: 1035-1040.
25. Gilmour SJ, Zarka DG, Stockinger EJ, Salazar MP, Houghton JM, et al. (1998) Low temperature regulation of the Arabidopsis CBF family of AP2 transcriptional activators as an early step in cold-induced COR gene expression. Plant J 16: 433-442.
26. Medina J, Bargues M, Terol J, Perez-Alonso M, Salinas J (1999) The Arabidopsis CBF gene family is composed of three genes encoding AP2 domain-containing proteins whose expression Is regulated by low temperature but not by abscisic acid or dehydration. Plant Physiol 119: 463-470.
27. Park J-E, Park J-Y, Kim Y-S, Staswick PE, Jeon J, et al. (2007) GH3-mediated Auxin Homeostasis Links Growth Regulation with Stress Adaptation Response in Arabidopsis. Journal of Biological Chemistry 282: 10036-10046.
28. Belin C, Megies C, Hauserova E, Lopez-Molina L (2009) Abscisic acid represses growth of the Arabidopsis embryonic axis after germination by enhancing auxin signaling. Plant Cell 21: 2253-2268.
29. Borevitz JO, Xia Y, Blount J, Dixon RA, Lamb C (2000) Activation Tagging Identifies a Conserved MYB Regulator of Phenylpropanoid Biosynthesis. The Plant Cell Online 12: 2383-2393.
30. Cominelli E, Gusmaroli G, Allegra D, Galbiati M, Wade HK, et al. (2008) Expression analysis of anthocyanin regulatory genes in response to different light qualities in Arabidopsis thaliana. Journal of Plant Physiology 165: 886-894.
31. Shen H, Cao K, Wang X (2008) AtbZIP16 and AtbZIP68, two new members of GBFs, can interact with other G group bZIPs in Arabidopsis thaliana. BMB Rep 41: 132-138.
32. Bieniawska Z, Espinoza C, Schlereth A, Sulpice R, Hincha DK, et al. (2008) Disruption of the Arabidopsis Circadian Clock Is Responsible for Extensive Variation in the Cold-Responsive Transcriptome. Plant Physiology 147: 263-279.

33. Umezawa LS, Yoshida R, Maruyama K, Yamaguchi-Shinozaki K, Shinozaki K (2004) SRK2C, a SNF1-related protein kinase 2, improves drought tolerance by controlling stress-responsive gene expression in Arabidopsis thaliana. Proceedings of the National Academy of Sciences of the United States of America 101: 17306-17311.
34. Yang LS, Poovaiah BW (2002) A Calmodulin-binding/CGCG Box DNA-binding Protein Family Involved in Multiple Signaling Pathways in Plants. Journal of Biological Chemistry 277: 45049-45058.
35. Nie H, Zhao C, Wu G, Wu Y, Chen Y, et al. (2012) SR1, a Calmodulin-Binding Transcription Factor, Modulates Plant Defense and Ethylene-Induced Senescence by Directly Regulating NDR1 and EIN3. Plant Physiology 158: 1847-1859.
36. Ouyang X, Li J, Li G, Li B, Chen B, et al. (2011) Genome-Wide Binding Site Analysis of FAR-RED ELONGATED HYPOCOTYL3 Reveals Its Novel Function in Arabidopsis Development. The Plant Cell Online 23: 2514-2535.
37. Lildballe DL, Pedersen DS, Kalamajka R, Emmersen J, Houben A, et al. (2008) The Expression Level of the Chromatin-Associated HMGB1 Protein Influences Growth, Stress Tolerance, and Transcriptome in Arabidopsis. Journal of Molecular Biology 384: 9-21.
38. Palma K, Zhao Q, Cheng YT, Bi D, Monaghan J, et al. (2007) Regulation of plant innate immunity by three proteins in a complex conserved across the plant and animal kingdoms. Genes & Development 21: 1484-1493.
39. Wei W, Huang J, Hao Y-J, Zou H-F, Wang H-W, et al. (2009) Soybean GmPHD-Type Transcription Regulators Improve Stress Tolerance in Transgenic Arabidopsis Plants. PLoS One 4: e7209.
40. Tsunoyama Y, Ishizaki Y, Morikawa K, Kobori M, Nakahira Y, et al. (2004) Blue light-induced transcription of plastid-encoded psbD gene is mediated by a nuclear-encoded transcription initiation factor, AtSig5. Proceedings of the National Academy of Sciences of the United States of America 101: 3304-3309.
41. Wei N, Deng XW (1992) COP9: a new genetic locus involved in light-regulated development and gene expression in arabidopsis. The Plant Cell Online 4: 1507-1518.
42. Yoshida LS, Nishimura N, Kitahata N, Kuromori LS, Ito LS, et al. (2006) ABA-hypersensitive germination3 encodes a protein phosphatase 2C (AtPP2CA) that strongly regulates abscisic acid signaling during germination among Arabidopsis protein phosphatase 2Cs. Plant Physiol 140: 115-126.
43. Saez A, Apostolova N, Gonzalez-Guzman M, Gonzalez-Garcia MP, Nicolas C, et al. (2004) Gain-of-function and loss-of-function phenotypes of the protein phosphatase 2C HAB1 reveal its role as a negative regulator of abscisic acid signalling. Plant J 37: 354-369.
44. Biswas KK, Ooura C, Higuchi K, Miyazaki Y, Van Nguyen V, et al. (2007) Genetic Characterization of Mutants Resistant to the Antiauxin p-Chlorophenoxyisobutyric Acid Reveals That AAR3, a Gene Encoding a DCN1-Like Protein, Regulates Responses to the Synthetic Auxin 2,4-Dichlorophenoxyacetic Acid in Arabidopsis Roots. Plant Physiology 145: 773-785.
45. Schumacher K, Vafeados D, McCarthy M, Sze H, Wilkins LS, et al. (1999) The Arabidopsis det3 mutant reveals a central role for the vacuolar H+–ATPase in plant growth and development. Genes & Development 13: 3259-3270.

46. Laxmi A, Pan J, Morsy M, Chen R (2008) Light Plays an Essential Role in Intracellular Distribution of Auxin Efflux Carrier PIN2 in <italic>Arabidopsis thaliana</italic>. PLoS One 3: e1510.
47. Allen GJ, Chu SP, Schumacher K, Shimazaki CT, Vafeados D, et al. (2000) Alteration of Stimulus-Specific Guard Cell Calcium Oscillations and Stomatal Closing in Arabidopsis det3 Mutant. Science 289: 2338-2342.
48. Kiyosue LS, Yamaguchishinozaki K, Shinozaki K (1993) Characterization of cDNA for a Dehydration-Inducible Gene That Encodes a CLP A, B-like Protein in Arabidopsis thaliana L. Biochemical and Biophysical Research Communications 196: 1214-1220.
49. Nakabayashi K, Ito M, Kiyosue LS, Shinozaki K, Watanabe A (1999) Identification of clp Genes Expressed in Senescing Arabidopsis Leaves. Plant and Cell Physiology 40: 504-514.
50. Simpson SD, Nakashima K, Narusaka Y, Seki M, Shinozaki K, et al. (2003) Two different novel cis-acting elements of erd1, a clpA homologous Arabidopsis gene function in induction by dehydration stress and dark-induced senescence. The Plant Journal 33: 259-270.
51. Peer WA, Hosein FN, Bandyopadhyay A, Makam SN, Otegui MS, et al. (2009) Mutation of the Membrane-Associated M1 Protease APM1 Results in Distinct Embryonic and Seedling Developmental Defects in Arabidopsis. The Plant Cell Online 21: 1693-1721.
52. Serino G, Su H, Peng Z, Tsuge LS, Wei N, et al. (2003) Characterization of the Last Subunit of the Arabidopsis COP9 Signalosome: Implications for the Overall Structure and Origin of the Complex. The Plant Cell Online 15: 719-731.
53. Cheng Y, Dai X, Zhao Y (2004) AtCAND1, A HEAT-Repeat Protein That Participates in Auxin Signaling in Arabidopsis. Plant Physiology 135: 1020-1026.
54. Estelle M, Somerville C (1987) Auxin-resistant mutants of Arabidopsis thaliana with an altered morphology. Molecular and General Genetics MGG 206: 200-206.
55. del Pozo JC, Estelle M (1999) The Arabidopsis cullin AtCUL1 is modified by the ubiquitin-related protein RUB1. Proceedings of the National Academy of Sciences 96: 15342-15347.
56. Moon J, Zhao Y, Dai X, Zhang W, Gray WM, et al. (2007) A New CULLIN 1 Mutant Has Altered Responses to Hormones and Light in Arabidopsis. Plant Physiology 143: 684-696.
57. Gilkerson J, Hu J, Brown J, Jones A, Sun LS-p, et al. (2009) Isolation and Characterization of cul1-7, a Recessive Allele of CULLIN1 That Disrupts SCF Function at the C Terminus of CUL1 in Arabidopsis thaliana. Genetics 181: 945-963.
58. Esteve-Bruna D, Pérez-Pérez JM, Ponce MR, Micol JL (2013) incurvata13, a Novel Allele of AUXIN RESISTANT6, Reveals a Specific Role for Auxin and the SCF Complex in Arabidopsis Embryogenesis, Vascular Specification, and Leaf Flatness. Plant Physiology 161: 1303-1320.
59. Thomann A, Lechner E, Hansen M, Dumbliauskas E, Parmentier Y, et al. (2009) Arabidopsis *CULLIN3* Genes Regulate Primary Root Growth and Patterning by Ethylene-Dependent and -Independent Mechanisms. PLoS Genet 5: e1000328.

60. Roberts D, Pedmale UV, Morrow J, Sachdev S, Lechner E, et al. (2011) Modulation of Phototropic Responsiveness in Arabidopsis through Ubiquitination of Phototropin 1 by the CUL3-Ring E3 Ubiquitin Ligase CRL3NPH3. The Plant Cell Online 23: 3627-3640.
61. Stowe-Evans EL, Harper RM, Motchoulski AV, Liscum E (1998) NPH4, a Conditional Modulator of Auxin-Dependent Differential Growth Responses in Arabidopsis. Plant Physiology 118: 1265-1275.
62. Harper RM, Stowe-Evans EL, Luesse DR, Muto H, Tatematsu K, et al. (2000) The NPH4 Locus Encodes the Auxin Response Factor ARF7, a Conditional Regulator of Differential Growth in Aerial Arabidopsis Tissue. The Plant Cell Online 12: 757-770.
63. Tatematsu K, Kumagai S, Muto H, Sato A, Watahiki MK, et al. (2004) MASSUGU2 Encodes Aux/IAA19, an Auxin-Regulated Protein That Functions Together with the Transcriptional Activator NPH4/ARF7 to Regulate Differential Growth Responses of Hypocotyl and Formation of Lateral Roots in Arabidopsis thaliana. The Plant Cell Online 16: 379-393.
64. Okushima Y, Overvoorde PJ, Arima K, Alonso JM, Chan A, et al. (2005) Functional Genomic Analysis of the AUXIN RESPONSE FACTOR Gene Family Members in Arabidopsis thaliana: Unique and Overlapping Functions of ARF7 and ARF19. The Plant Cell Online 17: 444-463.
65. Hardtke CS, Berleth LS (1998) The Arabidopsis gene MONOPTEROS encodes a transcription factor mediating embryo axis formation and vascular development. EMBO J 17: 1405-1411.
66. Schuetz M, Berleth LS, Mattsson J (2008) Multiple MONOPTEROS-Dependent Pathways Are Involved in Leaf Initiation. Plant Physiology 148: 870-880.
67. Ohashi-Ito K, Matsukawa M, Fukuda H (2013) An Atypical bHLH Transcription Factor Regulates Early Xylem Development Downstream of Auxin. Plant and Cell Physiology 54: 398-405.
68. Navarro C, Efremova N, Golz JF, Rubiera R, Kuckenberg M, et al. (2004) Molecular and genetic interactions between STYLOSA and GRAMINIFOLIA in the control of Antirrhinum vegetative and reproductive development. Development 131: 3649-3659.
69. Gonzalez D, Bowen AJ, Carroll TS, Conlan RS (2007) The Transcription Corepressor LEUNIG Interacts with the Histone Deacetylase HDA19 and Mediator Components MED14 (SWP) and CDK8 (HEN3) To Repress Transcription. Molecular and Cellular Biology 27: 5306-5315.
70. Gong Q, Li P, Ma S, Indu Rupassara S, Bohnert HJ (2005) Salinity stress adaptation competence in the extremophile Thellungiella halophila in comparison with its relative Arabidopsis thaliana. The Plant Journal 44: 826-839.
71. Lim G-H, Zhang X, Chung M-S, Lee DJ, Woo Y-M, et al. (2010) A putative novel transcription factor, AtSKIP, is involved in abscisic acid signalling and confers salt and osmotic tolerance in Arabidopsis. New Phytologist 185: 103-113.
72. He XJ, Mu RL, Cao WH, Zhang ZG, Zhang JS, et al. (2005) AtNAC2, a transcription factor downstream of ethylene and auxin signaling pathways, is involved in salt stress response and lateral root development. Plant J 44: 903-916.

73. Wu Y, Deng Z, Lai J, Zhang Y, Yang C, et al. (2009) Dual function of Arabidopsis ATAF1 in abiotic and biotic stress responses. Cell Res 19: 1279-1290.
74. Nelissen H, De Groeve S, Fleury D, Neyt P, Bruno L, et al. (2010) Plant Elongator regulates auxin-related genes during RNA polymerase II transcription elongation. Proceedings of the National Academy of Sciences 107: 1678-1683.
75. Zhou X, Hua D, Chen Z, Zhou Z, Gong Z (2009) Elongator mediates ABA responses, oxidative stress resistance and anthocyanin biosynthesis in Arabidopsis. The Plant Journal 60: 79-90.
76. Ueda A, Li P, Feng Y, Vikram M, Kim S, et al. (2008) The Arabidopsis thaliana carboxyl-terminal domain phosphatase-like 2 regulates plant growth, stress and auxin responses. Plant Molecular Biology 67: 683-697.
77. Yamamoto YY, Matsui M, Ang L-H, Deng X-W (1998) Role of a COP1 Interactive Protein in Mediating Light-Regulated Gene Expression in Arabidopsis. The Plant Cell Online 10: 1083-1094.
78. Zhao Y, Christensen SK, Fankhauser C, Cashman JR, Cohen JD, et al. (2001) A Role for Flavin Monooxygenase-Like Enzymes in Auxin Biosynthesis. Science 291: 306-309.
79. Cheng Y, Dai X, Zhao Y (2006) Auxin biosynthesis by the YUCCA flavin monooxygenases controls the formation of floral organs and vascular tissues in Arabidopsis. Genes & Development 20: 1790-1799.
80. Cheng Y, Dai X, Zhao Y (2007) Auxin Synthesized by the YUCCA Flavin Monooxygenases Is Essential for Embryogenesis and Leaf Formation in Arabidopsis. The Plant Cell Online 19: 2430-2439.
81. Marin E, Nussaume L, Quesada A, Gonneau M, Sotta B, et al. (1996) Molecular identification of zeaxanthin epoxidase of Nicotiana plumbaginifolia, a gene involved in abscisic acid biosynthesis and corresponding to the ABA locus of Arabidopsis thaliana. EMBO J 15: 2331-2342.
82. Gómez-Lim MA, Valdés-López V, Cruz-Hernandez A, Saucedo-Arias LJ (1993) Isolation and characterization of a gene involved in ethylene biosynthesis from Arabidopsis thaliana. Gene 134: 217-221.
83. Choi HI, Park HJ, Park JH, Kim S, Im MY, et al. (2005) Arabidopsis calcium-dependent protein kinase AtCPK32 interacts with ABF4, a transcriptional regulator of abscisic acid-responsive gene expression, and modulates its activity. Plant Physiol 139: 1750-1761.
84. Jayasekaran K, Kim KN, Vivekanandan M, Shin JS, Ok SH (2006) Novel calcium-binding GTPase (AtCBG) involved in ABA-mediated salt stress signaling in Arabidopsis. Plant Cell Rep 25: 1255-1262.
85. Nakashima K, Fujita Y, Kanamori N, Katagiri LS, Umezawa LS, et al. (2009) Three Arabidopsis SnRK2 protein kinases, SRK2D/SnRK2.2, SRK2E/SnRK2.6/OST1 and SRK2I/SnRK2.3, involved in ABA signaling are essential for the control of seed development and dormancy. Plant Cell Physiol 50: 1345-1363.
86. Boudsocq M, Droillard M-J, Barbier-Brygoo H, Laurière C (2007) Different phosphorylation mechanisms are involved in the activation of sucrose non-fermenting 1 related protein kinases 2 by osmotic stresses and abscisic acid. Plant Molecular Biology 63: 491-503.

87. Fujii H, Verslues PE, Zhu JK (2007) Identification of two protein kinases required for abscisic acid regulation of seed germination, root growth, and gene expression in Arabidopsis. Plant Cell 19: 485-494.
88. Cui Y, Ye J, Guo X, Chang H, Yuan C, et al. (2012) Arabidopsis casein kinase 1-like 2 involved in abscisic acid signal transduction pathways. Journal of Plant Interactions: 1-7.
89. Walsh TA, Neal R, Merlo AO, Honma M, Hicks GR, et al. (2006) Mutations in an Auxin Receptor Homolog AFB5 and in SGT1b Confer Resistance to Synthetic Picolinate Auxins and Not to 2,4-Dichlorophenoxyacetic Acid or Indole-3-Acetic Acid in Arabidopsis. Plant Physiology 142: 542-552.
90. Greenham K, Santner A, Castillejo C, Mooney S, Sairanen I, et al. (2011) The AFB4 Auxin Receptor Is a Negative Regulator of Auxin Signaling in Seedlings. Current biology : CB 21: 520-525.
91. Gray WM, Kepinski S, Rouse D, Leyser O, Estelle M (2001) Auxin regulates SCFTIR1-dependent degradation of AUX/IAA proteins. Nature 414: 271-276.
92. Dharmasiri N, Dharmasiri S, Estelle M (2005) The F-box protein TIR1 is an auxin receptor. Nature 435: 441-445.
93. Dai M, Zhang C, Kania U, Chen F, Xue Q, et al. (2012) A PP6-Type Phosphatase Holoenzyme Directly Regulates PIN Phosphorylation and Auxin Efflux in Arabidopsis. The Plant Cell Online 24: 2497-2514.
94. Dharmasiri N, Dharmasiri S, Weijers D, Lechner E, Yamada M, et al. (2005) Plant Development Is Regulated by a Family of Auxin Receptor F Box Proteins. Developmental cell 9: 109-119.
95. Weinl S, Kudla J (2009) The CBL–CIPK Ca2+-decoding signaling network: function and perspectives. New Phytologist 184: 517-528.
96. D'Angelo C, Weinl S, Batistic O, Pandey GK, Cheong YH, et al. (2006) Alternative complex formation of the Ca2+-regulated protein kinase CIPK1 controls abscisic acid-dependent and independent stress responses in Arabidopsis. The Plant Journal 48: 857-872.
97. Mizusawa K, Masuda S, Ohta H (2008) Expression profiling of four RelA/SpoT-like proteins, homologues of bacterial stringent factors, in Arabidopsis thaliana. Planta 228: 553-562.
98. Potuschak LS, Lechner E, Parmentier Y, Yanagisawa S, Grava S, et al. (2003) EIN3-Dependent Regulation of Plant Ethylene Hormone Signaling by Two Arabidopsis F Box Proteins: EBF1 and EBF2. Cell 115: 679-689.
99. Binder BM, Walker JM, Gagne JM, Emborg TJ, Hemmann G, et al. (2007) The Arabidopsis EIN3 Binding F-Box Proteins EBF1 and EBF2 Have Distinct but Overlapping Roles in Ethylene Signaling. The Plant Cell Online 19: 509-523.
100. Shi Y, Tian S, Hou L, Huang X, Zhang X, et al. (2012) Ethylene signaling negatively regulates freezing tolerance by repressing expression of CBF and type-A ARR genes in Arabidopsis. Plant Cell 24: 2578-2595.
101. Alonso JM, Hirayama LS, Roman G, Nourizadeh S, Ecker JR (1999) EIN2, a Bifunctional Transducer of Ethylene and Stress Responses in Arabidopsis. Science 284: 2148-2152.

102. Beaudoin N, Serizet C, Gosti F, Giraudat J (2000) Interactions between Abscisic Acid and Ethylene Signaling Cascades. The Plant Cell Online 12: 1103-1115.
103. Ghassemian M, Nambara E, Cutler S, Kawaide H, Kamiya Y, et al. (2000) Regulation of Abscisic Acid Signaling by the Ethylene Response Pathway in Arabidopsis. The Plant Cell Online 12: 1117-1126.
104. Shibuya K, Barry KG, Ciardi JA, Loucas HM, Underwood BA, et al. (2004) The Central Role of PhEIN2 in Ethylene Responses throughout Plant Development in Petunia. Plant Physiology 136: 2900-2912.
105. Wang Y, Liu C, Li K, Sun F, Hu H, et al. (2007) Arabidopsis EIN2 modulates stress response through abscisic acid response pathway. Plant Molecular Biology 64: 633-644.
106. Begheldo M, Manganaris GA, Bonghi C, Tonutti P (2008) Different postharvest conditions modulate ripening and ethylene biosynthetic and signal transduction pathways in Stony Hard peaches. Postharvest Biol Technol 48: 8-8.
107. Ding L, Pandey S, Assmann SM (2008) Arabidopsis extra-large G proteins (XLGs) regulate root morphogenesis. Plant J 53: 248-263.
108. Ahmad M, Jarillo JA, Smirnova O, Cashmore AR (1998) Cryptochrome blue-light photoreceptors of Arabidopsis implicated in phototropism. Nature 392: 720-723.
109. Blazquez MA, Ahn JH, Weigel D (2003) A thermosensory pathway controlling flowering time in Arabidopsis thaliana. Nat Genet 33: 168-171.
110. Motchoulski A, Liscum E (1999) Arabidopsis NPH3: A NPH1 photoreceptor-interacting protein essential for phototropism. Science 286: 961-964.
111. Trotta A, Wrzaczek M, Scharte J, Tikkanen M, Konert G, et al. (2011) Regulatory Subunit B′ γ of Protein Phosphatase 2A Prevents Unnecessary Defense Reactions under Low Light in Arabidopsis. Plant Physiology 156: 1464-1480.
112. Liu X, Zhu Y, Zhai H, Cai H, Ji W, et al. (2012) AtPP2CG1, a protein phosphatase 2C, positively regulates salt tolerance of Arabidopsis in abscisic acid-dependent manner. Biochemical and Biophysical Research Communications 422: 710-715.
113. Wang H, Lockwood SK, Hoeltzel MF, Schiefelbein JW (1997) The ROOT HAIR DEFECTIVE3 gene encodes an evolutionarily conserved protein with GTP-binding motifs and is required for regulated cell enlargement in Arabidopsis. Genes & Development 11: 799-811.
114. Oliviusson P, Heinzerling O, Hillmer S, Hinz G, Tse YC, et al. (2006) Plant Retromer, Localized to the Prevacuolar Compartment and Microvesicles in Arabidopsis, May Interact with Vacuolar Sorting Receptors. The Plant Cell Online 18: 1239-1252.
115. Parry G, Ward S, Cernac A, Dharmasiri S, Estelle M (2006) The Arabidopsis SUPPRESSOR OF AUXIN RESISTANCE proteins are nucleoporins with an important role in hormone signaling and development. Plant Cell 18: 1590-1603.
116. Sieburth LE, Muday GK, King EJ, Benton G, Kim S, et al. (2006) SCARFACE Encodes an ARF-GAP That Is Required for Normal Auxin Efflux and Vein Patterning in Arabidopsis. The Plant Cell Online 18: 1396-1411.

117. Møller SG, Kunkel LS, Chua N-H (2001) A plastidic ABC protein involved in intercompartmental communication of light signaling. Genes & Development 15: 90-103.
118. Kang J, Hwang JU, Lee M, Kim YY, Assmann SM, et al. (2010) PDR-type ABC transporter mediates cellular uptake of the phytohormone abscisic acid. Proc Natl Acad Sci U S A 107: 2355-2360.
119. Shi H, Ishitani M, Kim C, Zhu J-K (2000) The Arabidopsis thaliana salt tolerance gene SOS1 encodes a putative Na+/H+ antiporter. Proceedings of the National Academy of Sciences 97: 6896-6901.
120. Shi H, Quintero FJ, Pardo JM, Zhu J-K (2002) The Putative Plasma Membrane Na+/H+ Antiporter SOS1 Controls Long-Distance Na+ Transport in Plants. The Plant Cell Online 14: 465-477.
121. Quintero FJ, Ohta M, Shi H, Zhu J-K, Pardo JM (2002) Reconstitution in yeast of the Arabidopsis SOS signaling pathway for Na+ homeostasis. Proceedings of the National Academy of Sciences 99: 9061-9066.
