# Supplementary material for: A Bulk Segregant Gene Expression Analysis of a Peach Population Reveals Components of the Underlying Mechanism of the Fruit Cold Response
Source: PLoS One. 2014 Mar 5;9(3):e90706. doi: 10.1371/journal.pone.0090706 (PMC3944608; doi:10.1371/journal.pone.0090706)
Supplement: Methods S1 — Functional annotation of Chillpeach genes: functional categories, specific process/pathways, and relation to stress and hormones. (PDF) [file pone.0090706.s012.pdf]

Methods S1: Functional annotation of Chillpeach genes: functional categories, specific process/pathways, and relation to stress and hormones

ChillPeach unigenes were functionally annotated as indicated in Ogundiwin et al. [1]. The ChillPeach genes were classified into 34 distinct functional categories and 702 specific processes (Table S2) by extensively reviewing the literature and by searching in reference databases: PubMed [2], UniProt [3],prosite [4], BRENDA [5], TAIR [6], The Gene Index Project [7], KEEG [8,9], Plant Metabolic Network [10,11], and Plant Transcription Factor Database 2.0 [12].

To classify Chillpeach genes as stress and hormone responsive genes the AIG code of the *Arabidopsis* orthologues were used in a data mining strategy for interrogating the gene expression files from the following databases or papers:

Stress responsive genes:

(1) Cold, the ColdArrayDB (http://cold.stanford.edu/cgi-bin/data.cgi,) [13] a database that contains global expression profiles of *Arabidopsis* genes in response to cold. We use the same searching conditions as in [14].

(2) Cold, drought and salinity responsive genes, the results obtained with Affymetrix forward and reverse tiling arrays [15]

(3) darkness responsive genes the results obtained using a *Arabidopsis* Functional Genomics Consortium or 11K AFGC cDNA microarray by [16]

(4) Pathogen- virus responsive genes results obtained using 22K Affimetrix ATH1 GeneChip by [17].

Hormone related genes:

(1) Abscisic acid (ABA), auxin (Aux), brassinosteroid (Br), cytokinin (CK), ethylene (Et), gibberellin (GAs), jasmonic acid (JA) and salicylic acid (SA): Arabidopsis Hormone Database (http://ahd.cbi.pku.edu.cn, [18]) a comprehensive database based on

data from mutant studies, transgenic analysis, and gene ontology (GO) annotation for the hormones

(2) ABA, AUX, Br, CK, Et, GAs and JA responsive genes in the results obtained using Affimetrix ATH1 GeneChip as part of the AtGenExpress project by [19] and [20]

(3) ABA responsive genes using Arabidopsis Affymetrix tiling arrays identified by [15]

(4) ET-responsive genes by using cDNA-AFLP and a VIB Arabidopsis 6K cDNA microarray analysis as identified by [21]

References

1. Ogundiwin EA, Marti C, Forment J, Pons C, Granell A, et al. (2008) Development of ChillPeach genomic tools and identification of cold-responsive genes in peach fruit. Plant Mol Biol 68: 379-397.
2. PubMed [http://www.ncbi.nlm.nih.gov/pubmed].
3. Consortium TU (2012) Reorganizing the protein space at the Universal Protein Resource (UniProt). Nucleic Acids Research 40: D71-D75.
4. Sigrist CJA, de Castro E, Cerutti L, Cuche BA, Hulo N, et al. (2013) New and continuing developments at PROSITE. Nucleic Acids Research 41: D344-D347.
5. Schomburg I, Chang A, Placzek S, Söhngen C, Rother M, et al. (2013) BRENDA in 2013: integrated reactions, kinetic data, enzyme function data, improved disease classification: new options and contents in BRENDA. Nucleic Acids Research 41: D764-D772.
6. Lamesch P, Berardini TZ, Li D, Swarbreck D, Wilks C, et al. (2011) The Arabidopsis Information Resource (TAIR): improved gene annotation and new tools. Nucleic Acids Research.
7. Quackenbush J, Cho J, Lee D, Liang F, Holt I, et al. (2001) The TIGR Gene Indices: analysis of gene transcript sequences in highly sampled eukaryotic species. Nucleic Acids Research 29: 159-164.
8. Kanehisa M, Goto S (2000) KEGG: Kyoto Encyclopedia of Genes and Genomes. Nucleic Acids Research 28: 27-30.
9. Kanehisa M, Goto S, Sato Y, Furumichi M, Tanabe M (2012) KEGG for integration and interpretation of large-scale molecular data sets. Nucleic Acids Research 40: D109-D114.
10. Zhang P, Foerster H, Tissier CP, Mueller L, Paley S, et al. (2005) MetaCyc and AraCyc. Metabolic Pathway Databases for Plant Research. Plant Physiology 138: 27-37.
11. Zhang P, Dreher K, Karthikeyan A, Chi A, Pujar A, et al. (2010) Creation of a Genome-Wide Metabolic Pathway Database for Populus trichocarpa Using a New Approach for Reconstruction and Curation of Metabolic Pathways for Plants. Plant Physiology 153: 1479-1491.

12. Riano-Pachon D, Ruzicic S, Dreyer I, Mueller-Roeber B (2007) PlnTFDB: an integrative plant transcription factor database. BMC Bioinformatics 8: 42.
13. Vogel J, Zarka D, Van Buskirk H, Fowler S, Thomashow M (2005) Roles of the CBF2 and ZAT12 transcription factors in configuring the low temperature transcriptome of Arabidopsis. Plant J 41: 195 - 211.
14. Ogundiwin E, Marti C, Forment J, Pons C, Granell A, et al. (2008) Development of ChillPeach genomic tools and identification of cold-responsive genes in peach fruit. Plant Mol Biol 68: 379 - 397.
15. Matsui A, Ishida J, Morosawa T, Mochizuki Y, Kaminuma E, et al. (2008) Arabidopsis Transcriptome Analysis under Drought, Cold, High-Salinity and ABA Treatment Conditions using a Tiling Array. Plant and Cell Physiology 49: 1135-1149.
16. Kim BH, von Arnim AG (2006) The early dark-response in Arabidopsis thaliana revealed by cDNA microarray analysis. Plant Mol Biol 60: 321-342.
17. Ascencio-Ibáñez JT, Sozzani R, Lee T-J, Chu T-M, Wolfinger RD, et al. (2008) Global Analysis of Arabidopsis Gene Expression Uncovers a Complex Array of Changes Impacting Pathogen Response and Cell Cycle during Geminivirus Infection. Plant Physiology 148: 436-454.
18. Peng ZY, Zhou X, Li L, Yu X, Li H, et al. (2009) Arabidopsis Hormone Database: a comprehensive genetic and phenotypic information database for plant hormone research in Arabidopsis. Nucleic Acids Res 37: D975-982.
19. Goda H, Sasaki E, Akiyama K, Maruyama-Nakashita A, Nakabayashi K, et al. (2008) The AtGenExpress hormone and chemical treatment data set: experimental design, data evaluation, model data analysis and data access. Plant J 55: 526-542.
20. Nemhauser JL, Hong F, Chory J (2006) Different Plant Hormones Regulate Similar Processes through Largely Nonoverlapping Transcriptional Responses. Cell 126: 467-475.
21. De Paepe A, Vuylsteke M, Van Hummelen P, Zabeau M, Van Der Straeten D (2004) Transcriptional profiling by cDNA-AFLP and microarray analysis reveals novel insights into the early response to ethylene in Arabidopsis. The Plant Journal 39: 537-559.
