# Supplementary material for: A Bulk Segregant Gene Expression Analysis of a Peach Population Reveals Components of the Underlying Mechanism of the Fruit Cold Response
Source: PLoS One. 2014 Mar 5;9(3):e90706. doi: 10.1371/journal.pone.0090706 (PMC3944608; doi:10.1371/journal.pone.0090706)
Supplement: File S1 — Figures S1–S6. Figure S1. Frequency of the individuals with a given MI Index in the Pop-DG population.; Figure S2. The global gene expression analysis of the Chillpeach transcripts in response to cold storage; Figure S3. Projected MI correlated genes; Figure S4. 2D-HCA and PCAs for the genes in regulons ICE1, CBF1, HOS9, HOS15, ESK1, MYB-MYC, DREB2, AREB and ZF-NAC; Figure S5. The peach cold operons involved in the differential response between fruits S and LS; Figure S6. The differences between the microarray and qRT-PCR results in the magnitude of the expression levels for a selected number of genes. (PDF) [file pone.0090706.s013.pdf]

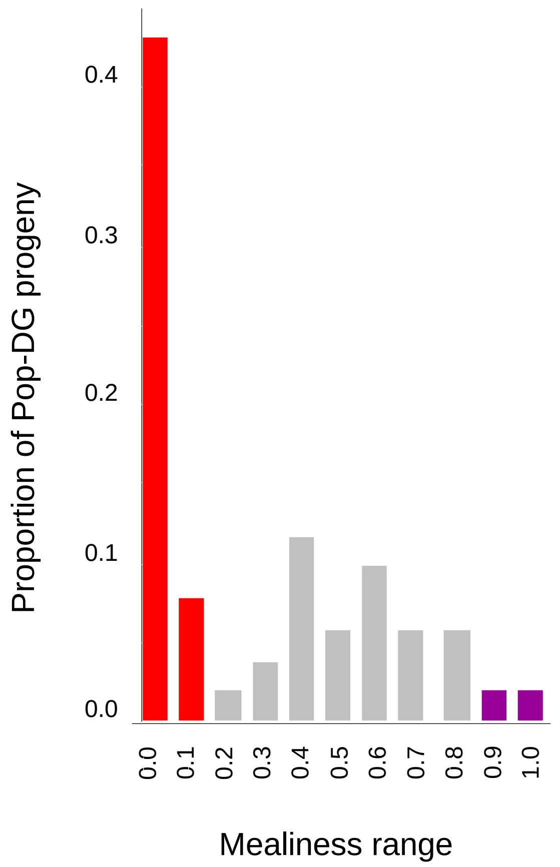

Figure S1. Frequency of the individuals with a given MI Index in the Pop-DG population. Symptoms scored after 1 week of cold storage plus shelf life ripening

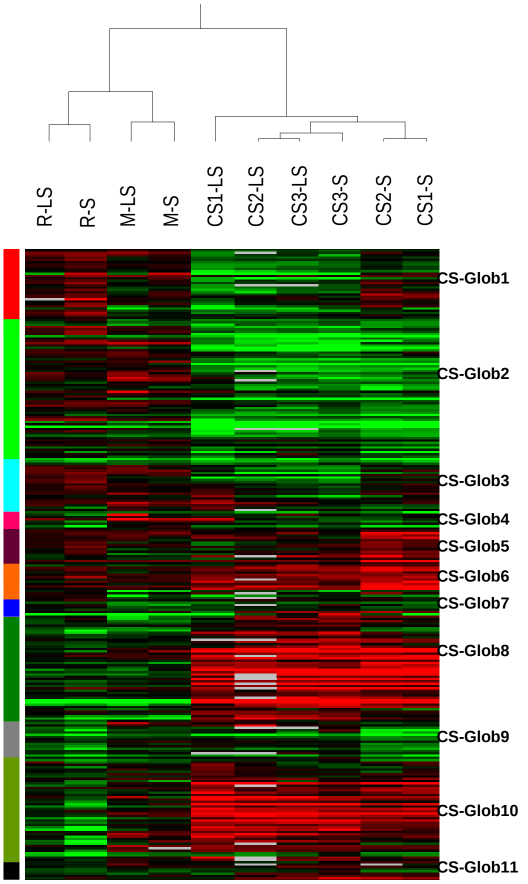

Figure S2. The global gene expression analysis of the Chillpeach transcripts in response to cold storage. Unsupervised bi-dimensional hierarchical clustering. Heat map of the expression values corresponding to the normalized means of three biological and three technical replicates. Color represents fold change (red upregulated/green down-regulated) in relation respect to a reference pool. Clustering of samples according to the expression values is shown on top M = mature fruits, R =mature with 2-4 days ripening at 20°C, CS1 = M + 1 week cold storage at 5°C, CS2 =M + 2 weeks cold storage at 5°C, CS3 = M + 3 weeks cold storage at 5°C

POSTIVE CORRELATION

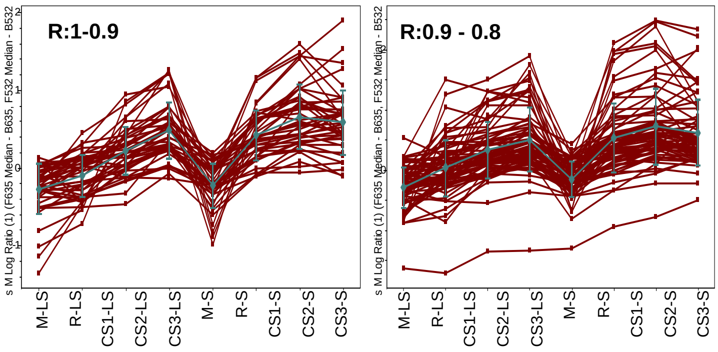

NEGATIVE CORRELATION

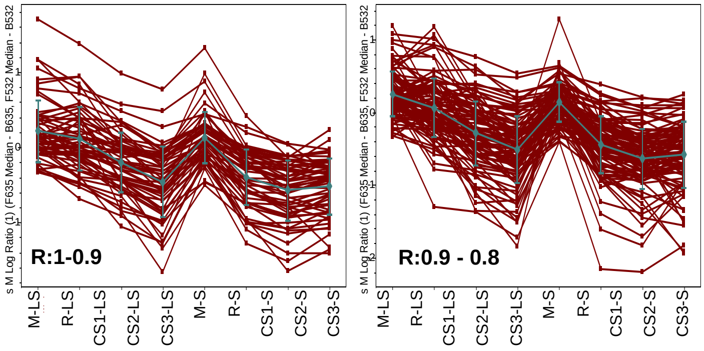

Figure S3. Projected MI correlated genes. The expression profiles of the genes in samples M and CS according to the mealiness phenotype. Phenotype-gene profile similarities were measured as Pearson correlation coefficients from the global dataset.

ICE1 regulon

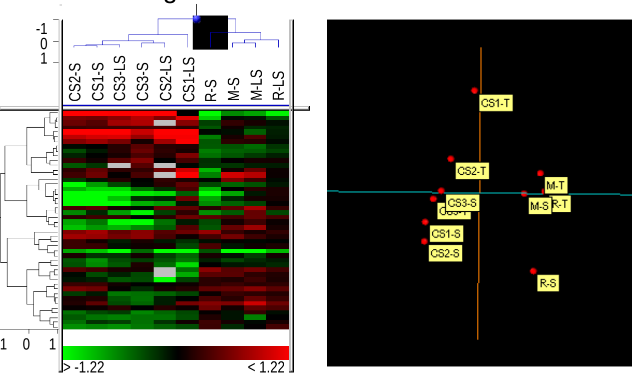

CBF regulon

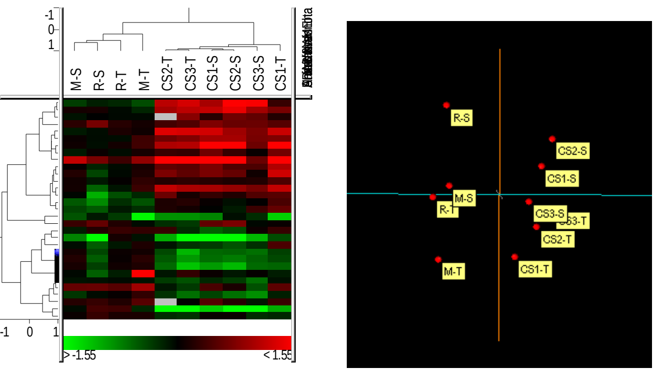

HOS9 regulon

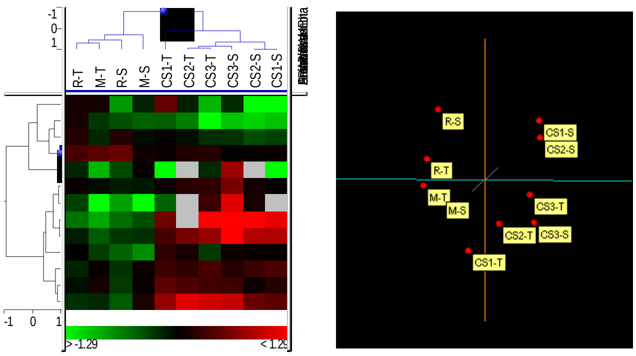

HOS15 regulon

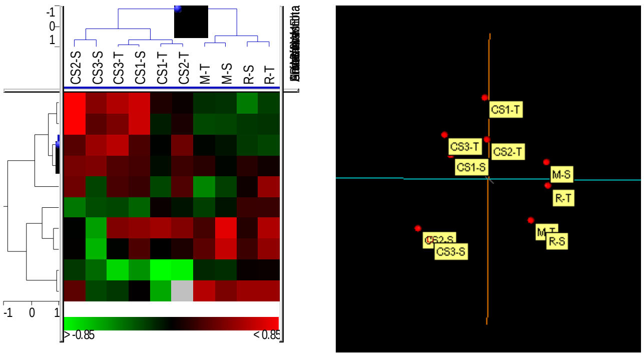

ESK1 regulon

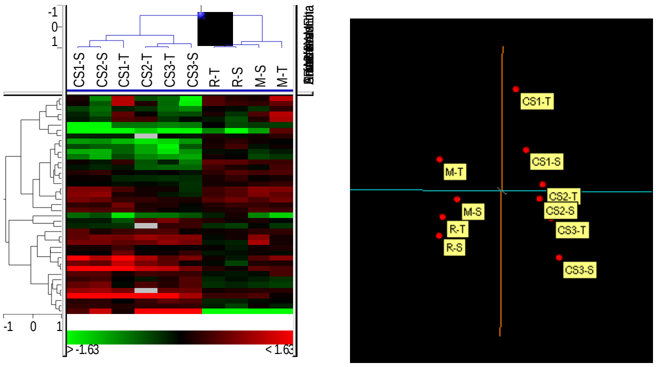

AREB regulon

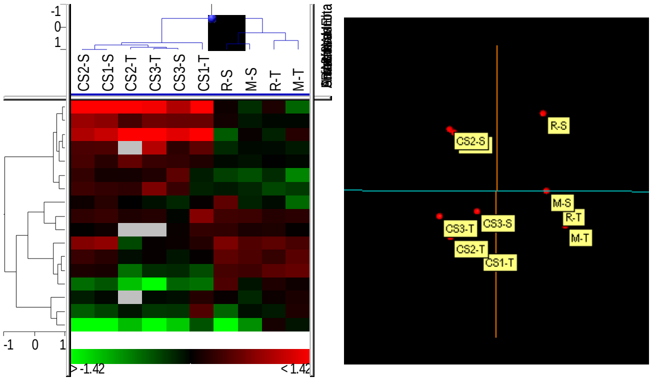

MYB-MYC regulon

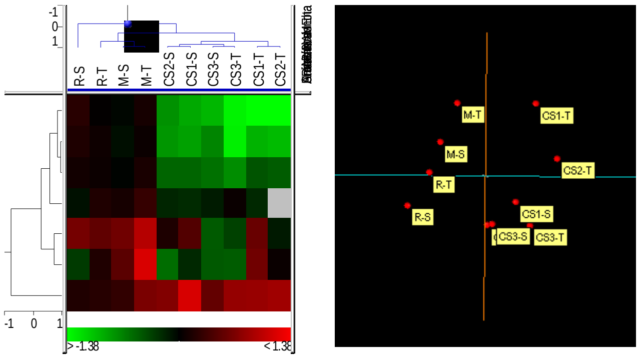

DREB2 regulon

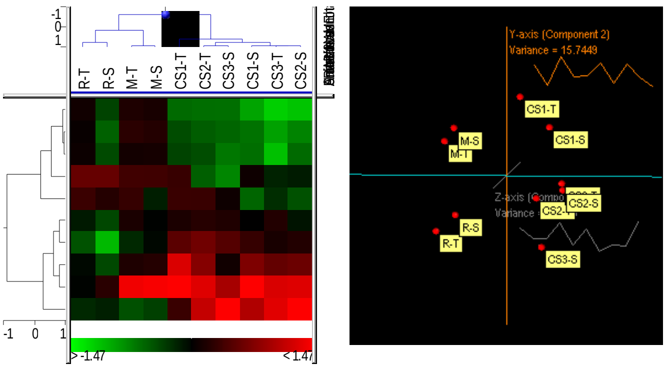

ZF-NAC regulon

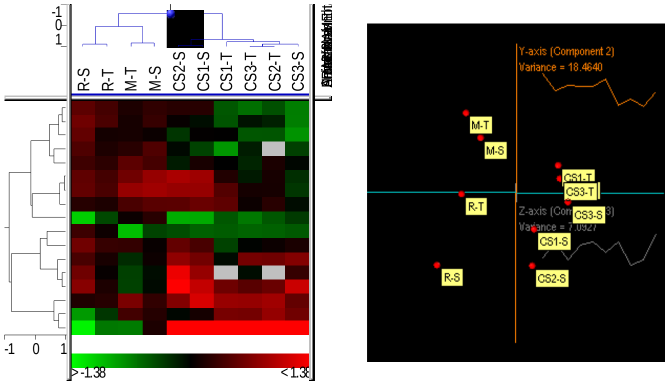

Figure S4. 2D-Hierarchical clusters and PCAs for the genes in regulons ICE1, CBF1, HOS9, HOS15, ESK1, MYB-MYC, DREB2, AREB and ZF-NAC. The PCA loading plots, PC1 vs. PC2 and 2D-hierarchical cluster. M = mature fruits, R = mature with 2-4 days ripening at 20°C, CS1 = M + 1 week cold storage at 5°C, CS2 = M + 2 weeks cold storage at 5°C, CS3 = M + 3 weeks cold storage at 5°C. S= sensitive, T =low senstive

A

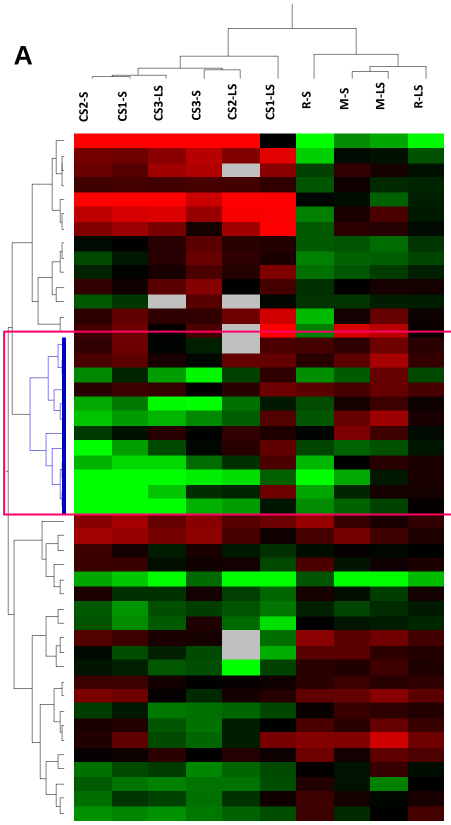

Chillpeach probe	Unigene annotation	AGI code	ARAB_NAME
PPN065F08	Sulfate transporter 3.1	AT3G51895	SULTR3;1
PPN055C11	Sucrose responsive element binding protein	AT5G67300	MYBR1
PPN051C10	ABI3-interacting protein 2, AIP2	AT5G20910	
PPN055B05	Similarity to heat shock transcription factor	AT3G24520	AT-HSFC1
PP1005B10	F19K23.17 protein	AT1G62250	
PP1000E05	Farnesylated protein	AT4G38580	ATFP6
PPN043A05	Putative desiccation protectant protein	AT1G01470	LEA14/LSR3
PPN019B02	AT5g18400/F20L16_120	AT5G18400	
PP1009C09	Expansin-like protein	AT3G45970	(ATEXLA1)
PPN016B02	Senescence-associated	AT2G17840	ERD7
PPN058A12	auxin-responsive protein-related,	AT2G45210	
PPN016B04	Putative fiber protein Fb1	AT3G10300	
PPN054A07	OSJNBb0039L24.13 protein	AT3G61260	
PPN048C10	Os01g0128300 protein	AT1G67080	ABA4
PPN059H05	Transcription factor ICE1	AT3G26744	ICE1
PPN064B12	Rust resistance gene ABC1041	AT2G28930	APK1B
PPN050B10	OSJNBa0086B14.2 protein	AT4G24380	
PPN046F08	Putative purple acid phosphatase	AT3G20500	PAP18
PPN058E05	Pyrrolidone carboxyl peptidase-like protein	AT1G56700	
PPN078A07	Calcium-binding protein	AT2G43290	MSS3
PPN044B01	Gene induced upon wounding stress	AT4G24220	VEP1
PP1000A02	Eukaryotic translation initiation factor 1b	AT5G54940	
PPN039G04	T17B22.3 protein	AT5G17350	
PPN054B03	CBF1	AT4G25480	CBF3/DREB1A
PPN054B07	Calcium-binding EF-hand	AT4G27280	
PP1004A05	SRC2	AT1G09070	SRC2
PPN017H05	Putative ZipA	AT4G32190	
PPN020F07	Gene induced upon wounding stress	AT4G24220	VEP1
PPN078A03	Cl-channel, voltage gated; IMP dehydrogenase related 1	AT5G33280	
PPN050H12	At5g47830	AT5G47830	
PPN001H09	YUP8H12R.39 protein	AT1G78990	
PPN078A02	Lipase, class 3	AT1G02660	
PPN051A12	Sugar transporter superfamily; MFS_1	AT5G26340	(MSS1)
PPN060D09	F6A14.6 protein	AT1G18850	
PPN004A11	Diacylglycerol kinase 1	AT5G07920	(DGK1)
PPN019C11	Translation initiation factor IF5	AT1G77840	
PPN006B03	Diacylglycerol kinase 1	AT5G07920	(DGK1)
PP1004F02	Cell division control protein 48 homolog E	AT3G53230	
PPN015D04	Metal-nicotianamine transporter YSL6	AT3G27020	(YSL6)
PPN062F07	Gb|AAF02142.1	AT3G17800	
PPN073E05	Purple acid phosphatase-like protein	AT3G20500	PAP18
PPN063G07	Alcohol dehydrogenase	AT1G77120	ATADH1
PPN035H06	Cys-3-His zinc finger protein	AT3G55980	SZF1
PP1009A02	CAMP response element binding (CREB) protein	AT4G34590	GBF6
PP1003A01	Soluble starch synthase	AT4G18240	SS4
PP1000G11	CAMP response element binding (CREB) protein	AT4G34590	GBF6
PPN032A06	T4P13.28 protein	AT3G58790	GAUT15

B

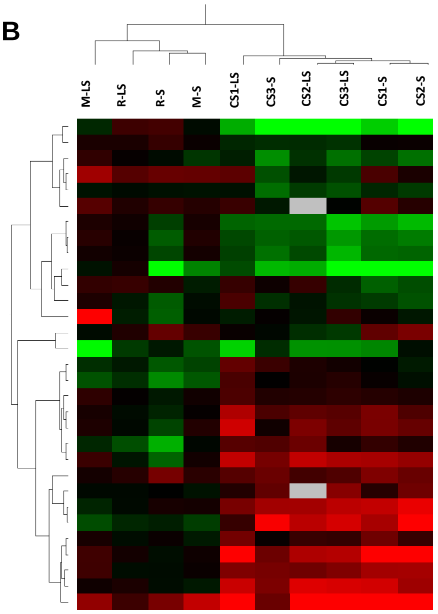

Chillpeach probe	Unigene annotation	AGI code	ARAB_NAME
PP1004E10	Cinnamyl-alcohol dehydrogenase 1	AT4G34230	CAD5
PPN060B12	Pyrroline-5-carboxylate synthetase	AT3G55610	P5CS2
PPN054D12	Legumain precursor	AT1G62710	beta-VPE
PPN063G07	Alcohol dehydrogenase	AT1G77120	ATADH1
PPN011D01	CDNA FLJ10846 fis, clone NT2RP4001373	AT4G21570	
PPN059H05	Transcription factor ICE1	AT3G26744	ICE1
PPN042F10	C2.6 protein	AT3G53990	
PPN023C01	Early nodulin ENOD18	AT3G53990	
PP1000A06	Early nodulin ENOD18	AT3G53990	
PPN054B03	CBF1	AT4G25480	CBF3/DREB1A
PPN016D07	Heat shock cognate 70 kDa protein 2	AT3G12580	HSP70
PPN025F02	AtHVA22a	AT1G74520	AtHVA22a
PP1006B11	Expansin precursor	AT1G69530	ATEXPA1
PPN021B11	Casein kinase	AT3G23340	CKL10
PP1001A05	Pyruvate decarboxylase 1	AT4G33070	Pdc1
PPN016B02	Senescence-associated	AT2G17840	ERD7
PP1000C09	Putative ripening-related protein	AT5G62350	FL5-2I22
PPN043F07	Alpha-glucan water dikinase, chloroplast precursor	AT1G10760	sex1
PPN033F01	Serpin-like protein	AT1G47710	
PPN043A05	Putative desiccation protectant protein	AT1G01470	LEA14/LSR3
PPN073G09	Early nodulin ENOD18	AT3G53990	
PP1000E05	Farnesylated protein	AT4G38580	ATFP6
PPN017H05	Putative ZipA	AT4G32190	
PPN067H06	WD40-like	AT4G04920	SFR6
PPN073E12	Protein phosphatase 2C-like	AT5G27930	PP2C
PPN072F12	Heat shock 70 kDa protein	AT3G12580	HSP70
PP1005G04	Serpin-like protein	AT1G47710	
PP1005C02	CD9/CD37/CD63 antigen	AT3G45600	TET3
PP1006E03	Os08g0152800 protein	AT1G33230	
PP1004H05	ABA-responsive protein-like	AT5G13200	
PPN055E08	Arabidopsis thaliana, mRNA sequence	AT2G27830	

C

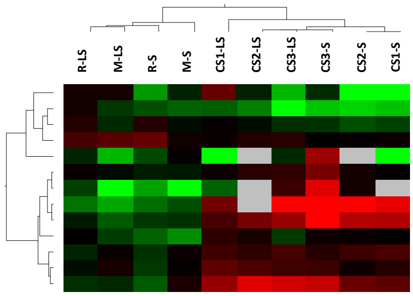

Chillpeach probe	Unigene annotation	AGI code	ARAB_NAME
PPN054B07	Calcium-binding EF-hand	AT4G27280	
PPN059B10	Os01g0186900 protein	AT4G29780	
PPN067A05	Copine-like protein	AT5G61900	BON1
PPN035H06	Cys-3-His zinc finger protein	AT3G55980	SZF1
PPN028G09	Putative calcium-LSransporting ATPase 13	AT3G22910	
PPN040A05	Putative calcium-LSransporting ATPase 13	AT3G22910	
PPN058B08	0.00E+00	AT1G13340	
PPN041C03	Gb|AAC98457.1	AT5G60800	
PPN071E03	F21O3.6 protein	AT2G39650	
PPN051E04	Avr9/Cf-9 rapidly elicited protein 284	AT2G30020	
PPN064C10	Gb|AAC98457.1	AT5G60800	
PPN039A03	ATFP3	AT5G63530	FP3
PPN037H08	Putative alanine acetyl transferase	AT2G32030	

Figure S5. The peach cold operons involved in the differential response between fruits S and T. The bidimensional hierarchical cluster expression values for the 46 genes described as in regulon ICE1 (A) for the 31 genes described as in regulon CBF (B) and for the 13 genes described in regulon HOS9.

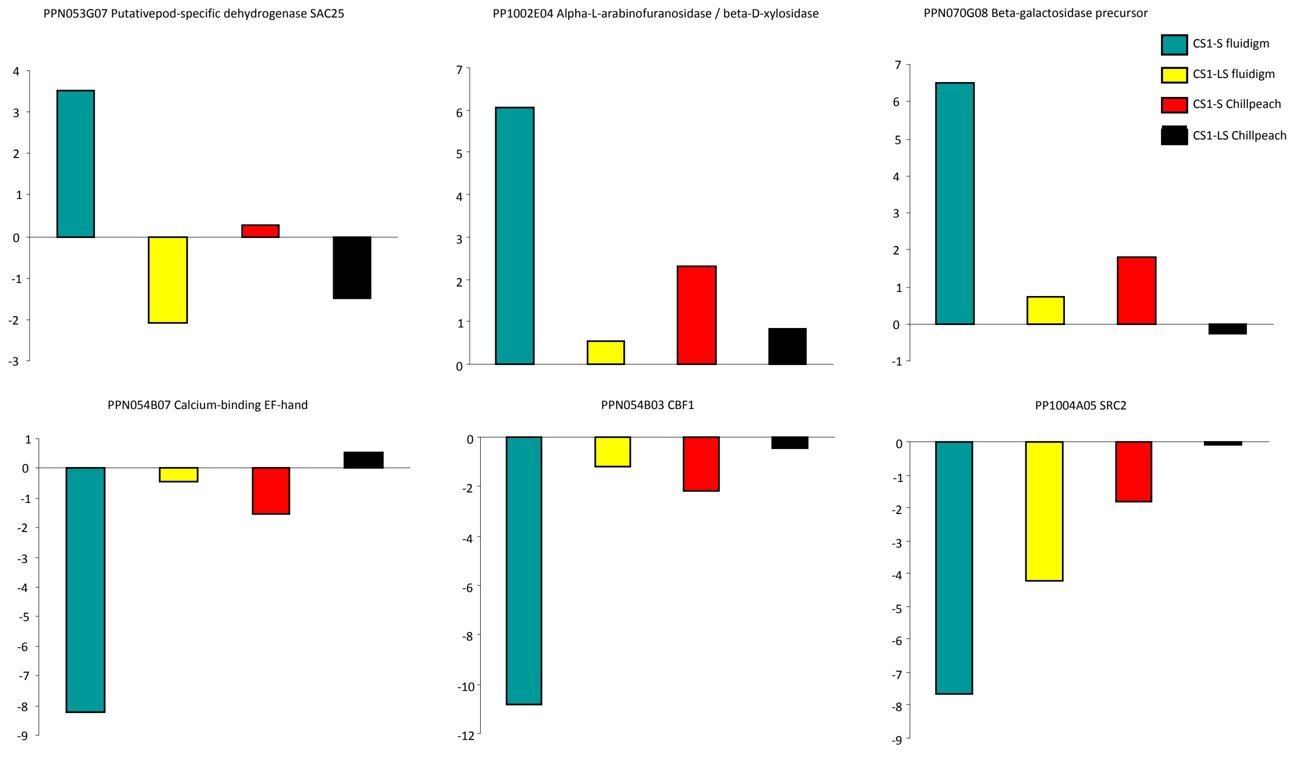

Figure S6 The differences between the microarray and qRT-PCR results in the magnitude of the expression levels for a selected number of genes. The y-axes represents the normalized fold expression change respect to the levels in the reference pool.
